# Links between host genetics, metabolism, gut microbiome and amoebic gill disease (AGD) in Atlantic salmon

**DOI:** 10.1186/s42523-022-00203-x

**Published:** 2022-09-15

**Authors:** Patrick Schaal, Bachar Cheaib, Joshka Kaufmann, Karl Phillips, Liz Ryder, Phil McGinnity, Martin Llewellyn

**Affiliations:** 1 Institute of Biodiversity, Animal Health and Comparative Medicine, University of Glasgow, Glasgow, UK; 2 School of Biological, Earth and Environmental Sciences, University College Cork, Cork, Ireland; 3 Marine Institute, Newport, Ireland

## Abstract

**Background:**

Rapidly spreading parasitic infections like amoebic gill disease (AGD) are increasingly problematic for Atlantic salmon reared in aquaculture facilities and potentially pose a risk to wild fish species in surrounding waters. Currently, it is not known whether susceptibility to AGD differs between wild and farmed salmon. Wild Atlantic salmon populations are declining and this emerging disease could represent an additional threat to their long-term viability. A better understanding of how AGD affects fish health is therefore relevant for the accurate assessment of the associated risk, both to farming and to the well-being of wild populations. In this study, we assessed the impact of natural exposure to AGD on wild, hybrid and farmed post-smolt Atlantic salmon reared in a sea farm together under common garden conditions.

**Results:**

Wild fish showed substantially higher mortality levels (64%) than farmed fish (25%), with intermediate levels for hybrid fish (39%) suggesting that AGD susceptibility has an additive genetic basis. Metabolic rate measures representing physiological performance were similar among the genetic groups but were significantly lower in AGD-symptomatic fish than healthy fish. Gut microbial diversity was significantly lower in infected fish. We observed major shifts in gut microbial community composition in response to AGD infections. In symptomatic fish the relative abundance of key taxa *Aliivibrio*, *Marinomonas* and *Pseudoalteromonas* declined, whereas the abundance of* Polaribacter* and *Vibrio* increased compared to healthy fish.

**Conclusions:**

Our results highlight the stress AGD imposes on fish physiology and suggest that low metabolic-rate fish phenotypes may be associated with better infection outcomes. We consider the role increased AGD outbreak events and a warmer future may have in driving secondary bacterial infections and in reducing performance in farmed and wild fish.

**Supplementary Information:**

The online version contains supplementary material available at 10.1186/s42523-022-00203-x.

## Background

Atlantic salmon aquaculture is one of the largest and most profitable fish production industries worldwide [1]. Large scale production accompanied by high fish densities has led to ecological and economical challenges and increased the pressure to make aquaculture more sustainable [2, 3]. Whilst the salmonid aquaculture industry is undergoing rapid expansion, fish are increasingly prone to numerous stressors associated with pen-rearing in the marine environment. Anthropogenic stressors include behavioural and physiological stress induced by high stocking densities [4], the use of terrestrial protein and lipid sources that impact gut health [5–7] and enhanced handling stress associated with the treatment of fish for several important parasitic diseases [8, 9]. Biological stressors include a variety of plankton-borne threats, especially sea lice (e.g., parasitic copepods; [10, 11]), micro-jellyfish and harmful algal blooms [12] and amoebic gill disease [13]. The presence of large volumes of farmed fish along North Atlantic coastlines also impacts wild salmon populations via epizootics and genetic introgression from farm stocks [14, 15].

Amoebic gill disease (AGD) is increasingly problematic for the marine phase of salmonid aquaculture globally [13]. In recent years, this disease has caused substantial economic losses on Scottish and Irish salmon farms amounting to millions of British Pound Sterling [16, 17]. AGD is caused by the ectoparasitic protozoan *Neoparamoeba perurans*, which colonises fish’s gill epithelium inducing lamellar fusion resulting in anorexia, increased ventilation rates and eventually death if not treated [18, 19]. Whilst AGD infections are well documented in farmed salmonids little is known about their effect on wild fish [20]. In the wild, predators may rapidly eliminate affected wild fish making an assessment difficult [21]. There is growing evidence of significant transmission of infectious diseases from farmed fish to wild fish, which occurs either due to farmed escapes or by wild fish being in close proximity to aquaculture pens while feeding or in process of migrating [22, 23]. Climate change accompanied by rising water temperatures make AGD outbreaks more likely and therefore a pressing subject for future research [13, 24]. It is especially important to assess the sublethal effects of AGD infections, their impact on fish physiology and the potential impact of AGD on wild salmonids in particular.

Two important physiological measures of interest are standard metabolic rate (SMR) and maximum metabolic rate (MMR). SMR is the minimal maintenance metabolic rate of an ectotherm in a post-absorptive and inactive state [25]. MMR describes the upper limit to an organism’s ability to take up oxygen [26]. The difference between MMR and SMR is the aerobic scope (AS), which represents the amount of energy available for activities like locomotion, feeding, digestion, growth, and reproduction [27, 28]. Host metabolic rate has strong ecological relevance [29] by impacting fish growth and survival [30]. Variation in metabolic rate measures between individuals are likely to have fitness consequences [31]. For example, fish with high metabolic rates might be able to grow faster in favourable living conditions but struggle during times of food scarcity or stress (e.g., parasitic infections) due to their high maintenance costs [32–34]. High SMR individuals have also been shown to digest meals faster, potentially resulting in higher food intake and consequentially a greater growth potential [35]. Since farmed fish show an upregulation of genes associated with energy metabolism [36], have bigger food intake and also tend to be bigger than their wild counterparts [14] one might assume underlying differences in metabolic phenotype between the groups. It is known that AGD infections compromise gill functions, resulting in lower MMR values [37]. However, whether there is a differential impact of AGD on individuals with distinct genetic backgrounds and metabolic rates has yet to be determined.

Microbiomes can be defined as microbial communities, their genomes and surrounding environmental conditions in well-defined habitats [38, 39]. Gut microbial communities are known to impact host metabolism [40], growth [41], behaviour [42], immune response [43] and pathogen defence [44]. Microbial communities are shaped by selective [45–47] and stochastic processes [48, 49]. Different gut compartments e.g., the pyloric caeca (PC) or midgut (MG) have distinct microbial profiles [50, 51] which might be connected to their different functionalities [52–54]. Correlations between host phenotype and microbiome are commonly reported [55] with stress being an important driver of microbiome change in aquaculture systems [56]. In addition, host-associated microbial communities can be a source of opportunistic pathogens associated with parasite infection [57]. Another poorly studied aspect of fish microbiome ecology is the effect of fasting [58, 59]. It is not uncommon for fish in the wild to spend prolonged periods of time without food, e.g. due to seasonal changes in food availability [60]. It is a widespread practice to starve fish prior to major farming operations like crowding, pumping, delousing, and transportation [61]. Also, fish will often lose appetite on transfer from freshwater to the sea and may take some time acclimatising and resuming feeding in the new environment [62]. We hypothesize that deteriorating host health, induced by external stressors like parasitic infections also affects gut microbial community composition. Hence, tracking host-associated microbiota can provide clues to changing host physiological status and performance as a result of primary parasitic and possible secondary infections.

In the current study, we aim to understand the impact of natural exposure of common garden-reared wild, hybrid and farmed fish to amoebic gill disease. Alongside classical measures of the fish condition and AGD-status, we also monitored the impact of AGD on host respiratory physiology across the different fish cohorts. Finally, we also explored the impact of AGD infection on the host’s gut microbiology.

## Methods

### Experimental setup

**Fig. 1:**
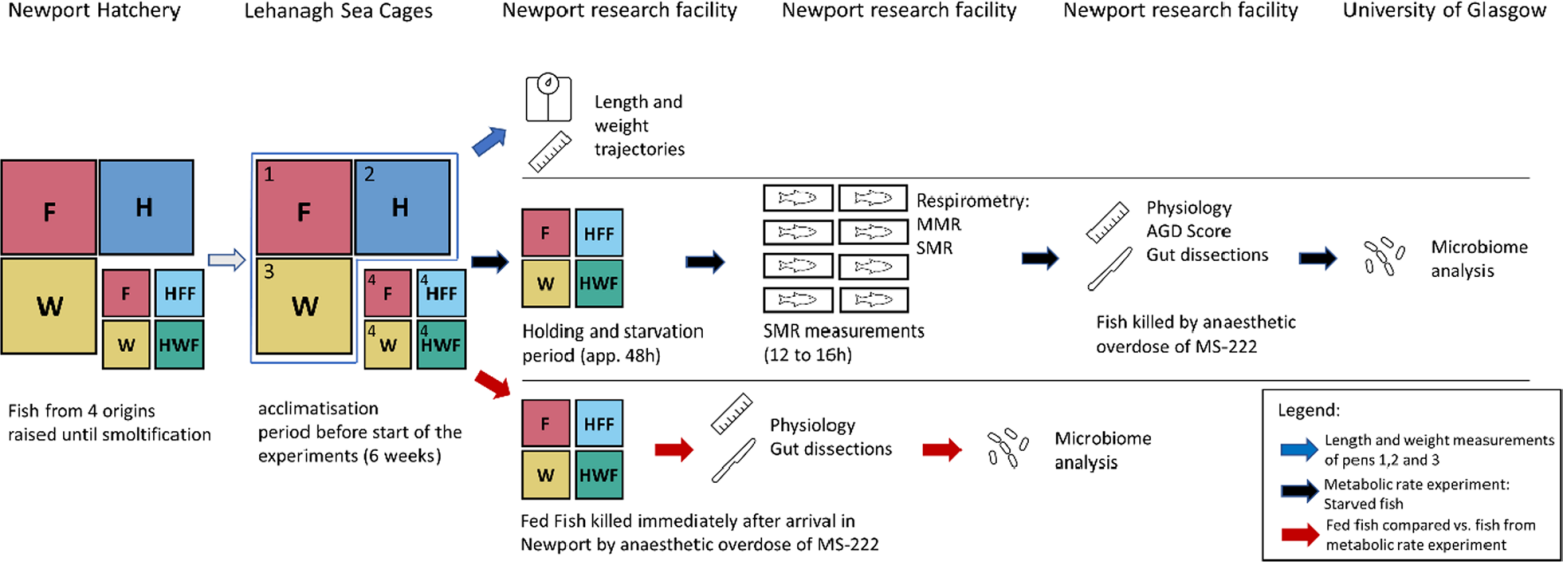
Flow chart of the experimental setup. Fish from four genetic backgrounds were used: Farmed (F), wild (W) and their reciprocal hybrids named after the origin of the mother (hybrid farmed female (HFF) and hybrid wild female (HWF)). Fish from pens 1–3 were used for length and weight trajectories (blue arrow). PIT tagged fish from pen 4 were used for the respirometry experiment and microbiome analysis (black arrow). In addition, we compared starved fish from the metabolic rate experiment with recently fed fish at two different sampling timepoints T0 (day 0 of respirometry experiment) and T1 (day 14 of the respirometry experiment) (red arrow)

Atlantic salmon of four different genetic origins were raised in hatchery ponds at the Marine Institute research facility at Newport, County Mayo, Ireland. The four groups consisted of fish from the progeny of a commonly reared farmed strain (F), wild fish sourced from the Burrishoole river in the west of Ireland (W) and their reciprocal hybrid progeny of farm and wild parents (HFF and HWF, respectively). The fish were fed ad libitum on a diet of pellets produced by Skretting Nutra Olympic (Cheshire, UK) during freshwater rearing. Individuals were tagged with passive transponder tags (PIT tags) for later identification. On smoltification, May 2019, the four groups of smolts were transferred to a sea pen at the Marine Institutes Lehanagh Pool research site, Ireland (Cashel Bay, 53.401116, − 9.819287). Within the pen were four sentinel pens (4 × 4 m). A subset of farmed, wild and hybrid fish groups was introduced into each of three of the sentinel pens, one for each of farmed (app. 380 fish), wild (app. 360 fish) and combined hybrid groups (app. 700 fish). The fish in these pens were used for the tracking of length and weight trajectories of the groups throughout the study. Twenty fish of each cohort (40 in the case of hybrids) were measured weekly. The fourth sentinel pen contained a mixture of PIT tagged fish representing all four experimental groups (F: n = 207; HFF: n = 204; HWF: n = 194; W: n = 198)). Post smolts in the sea pen were fed a maintenance diet of Ewos 75 pellets produced by Cargill (MN, USA) on a five-day cycle. Mortality per genetic group in the mixed sentinel pen was assessed by comparing the fish counts from the start and end of the experiment. After a settlement period of six weeks at sea 16 fish (four fish of each genetic background) were transferred from the mixed (4th) sentinel pen twice a week to the Newport research facility for metabolic rate measurements and subsequent dissections of organs and guts (Fig. 1). In total, we had 11 sampling days over the course of 49 days. During this time some fish showed signs of AGD, especially at the later stages of the experiment. Gut microbiome analysis was carried out at the University of Glasgow. An overview of sample identification number, sampling days (the date when fish were caught), water temperatures and processing days (the day fish were dissected) is shown in Additional File 1: Table S1.

### Metabolic rate measurements

The oxygen consumption (ṀO_2_) of individual fish was determined using automated intermittent respirometry as described previously [63, 64] and using best practices outlined in [25, 65, 66].

After transfer to the Marine Institute respirometry laboratory, fish were placed in holding tanks for 24 h. Natural hiding spots were provided to reduce fish stress. Fish were not fed for a period of 48 h to ensure that they were in a post-absorptive state required for accurate metabolic rate measurements. To determine the fish’s maximum metabolic rate (MMR) fish were chased in a bucket for 60 s and immediately transferred to the respirometry setup [25]. The setup consisted of respirometry chambers (rectangular sealable plastic boxes with a total volume of 2 L), which were contained within an oxygenated and temperature-controlled reservoir filled with seawater. Oxygen concentration in the respirometry chambers was measured constantly by using fibre-optic oxygen meter probes (FireStingO2; Pyro Science GmbH, Aachen, Germany) and accompanying software (Pyro Oxygen Logger; Pyro Science GmbH, Germany). Automated flush pumps refreshed the water in the respirometers for 6 min in every 10 min period and ṀO_2_ was calculated from the decline in oxygen concentration in the respirometers between flush cycles [67].

### Metabolic rate data analysis

Data obtained from the respirometry measurements was uploaded into R [68]. Analysis was carried out using the R packages respirometry [69] and custom functions. For MMR analysis we first plotted the slope of the decline in MO_2_ over time. Then, we assessed the start and end times of the measurement cycle visually for each chamber (fish) individually. To provide clean slopes, we chose to cut the observed slope 5 s after the maximum MO_2_ value and 5 s before the lowest MO_2_ value of the measurement cycle.

SMR was calculated for each fish by calculating the quantiles that assign 20% of the data below mean SMR (q_0.2_). Mean SMR was assessed from slopes that had an R_2_ of at least 0.8 (r_2min_ = 0.8). This method accounts for periods of high activity by the fish throughout the measurement period and was determined as per [25] as best estimate for SMR, when fish activity levels can’t be observed visually.

Metabolic rates were size corrected using general linear models with body mass (weight) as covariate (ANCOVA) [70]. This allows a direct comparison of individuals of different masses and consequently accounts for any differences in metabolism arising from size differences found among fish from the different groups. To linearise the data, metabolic rate and body mass measures were log10-transformed. To maintain comparability within the study 19 out of 145 metabolic rate measurements were excluded from the analysis due to inconsistencies in fish handling time or equipment set-up errors.

### Gut microbiome collection and AGD scoring

After SMR measurements fish were euthanized by an anaesthetic overdose of methane tricaine sulphonate (MS-222, 300 mg/L, FVG, Ireland). Histological AGD lesion assessment is known to be good qualitative tool for AGD scoring [71]. Therefore, fish gills were visually examined for a potential AGD infection and AGD severity was determined by using the scoring system described by [72] (Table 1).

**Table 1:** AGD scoring system [72]

AGD score	AGD severity	Description
0	Clear	No sign of infection and healthy red colour (non-symptomatic)
1	Very light	1 white spot, light scarring or undefined necrotic streaking
2	Light	2–3 white spots/small mucus patch
3	Moderate	Established thickened mucus patch/ white spot groupings up to 20% of gill area
4	Advanced	Established lesions covering up to 50% of gill area
5	Heavy	Extensive lesions covering most of the gill surface (over 50%)

Fish were classified depending on visual inspection of the gills. White spots and mucus patches on the gills are typical indicators of an AGD infection

Wet weight (g) and fork length (mm) were measured. Fulton’s condition factor (K) was calculated as follows [73]:

$${\text{K}} = {1}00 \times {\text{weight}}\;{\text{(g)/length}}\;({\text{cm}})^{{3}}$$

Fish were dissected aseptically via an incision along the ventral side. Organ weights and gut length were measured, and sections of the gut compartments pyloric caeca and mid-gut were separated, put into cryotubes and immediately placed on dry ice. Samples were subsequently stored at − 80 °C and later shipped on dry ice to the University of Glasgow for microbiome analysis.

Each dissected fish was reweighed without its gut and placed into a drying oven at 60 °C for 72 h before measuring its dry weight. The wet mass (g, excluding the gut) and dry mass (g) was then used to determine the % water content as follows:

$$\% {\text{Water content}} = {1}00\,(({\text{wet mass}}{-}{\text{dry mass}}){\text{/wet mass}})$$

% water content was also used to estimate the fat content of the fish, since it has previously been shown that there is a strong negative correlation between % water content and % fat content [74].

### Environmental controls and determination of feeding status

At each sampling date environmental samples were taken to identify bacteria in the water column. Sterile water bottles (1.5 L) were used to collect seawater at a depth of app. one meter inside sentinel cage four. The seawater was filtered in a sterile environment using 0.2 µm filters (Whatman, Chicago, IL, USA). After filtration the filters were placed into cryotubes, immediately placed on dry ice and stored at − 80 °C prior to transportation to the University of Glasgow and microbiome profiling.

To account for the influence of food withdrawal and holding times on fish gut microbiome community composition, recently fed control fish were sampled in addition to the regular sampling procedure. Eight fish (two fish of each genetic background) were sampled right after the 6 weeks acclimatisation period, which equals the start of the metabolic rate experiments. Those fish were labelled “T0Fed”. Fourteen days later another 8 recently fed fish (again two fish of each genetic background) were sampled and labelled “T1Fed”. At each of the two timepoints the gut microbial community composition of the fed fish was compared with 8 starved fish. Those fish were sampled (caught) at the same day but were starved for 48 h during metabolic rate measurements. These samples are labelled T0Starved and T1Starved, respectively. All fish in this control study were asymptomatic for AGD.

### Microbial DNA extraction and NGS library preparation

The DNA extraction and NGS library preparation protocols used were based on methods established and summarized in [49, 50].

To extract bacterial DNA from gut samples, the frozen gut tissue (200 mg) and filter papers were cut up into pieces using sterilized equipment and DNA was extracted using the QIAamp DNA Stool Mini Kit (Qiagen, Valencia, CA, USA) according to the manufacturer’s protocol [75].

Extracted DNA was amplified using primers targeting the V1 hypervariable 16S rDNA region [76]. V1 was chosen over V4 because it showed less cross-contamination with salmon DNA [49, 77]. Amplification of the target region was achieved by using tagged barcodes 27F and 338R at a concentration of 1 pM for each primer. Primer sequences are shown in Additional file 1: Table S1. The reaction mix contained a total volume of 15 μL and consisted of 0.7 μL of each internal forward and reverse primer, 7 μL of Q5 Hot Start High-Fidelity 2X Master Mix (New England Biolabs Ltd, UK) and 1 μL of DNA template. PCR conditions were initial denaturation at 95 °C for 10 min; 30 cycles at 95 °C for 30 s, 55 °C for 30 s and 72 °C for 30 s; and a final elongation step of 72 °C for 10 min. First round PCR products were then used for a subsequent second round of PCR in which external multiplex identifiers (barcodes) were added. The second round PCR reaction mix contained a total volume of 25 μL and consisted of 1.25 μL of external revers primer (10 μM), 1.25 μL external forward primer (10 μM), 12.5 μL of Q5 Hot Start High-Fidelity 2X Master Mix (New England BioLabs Ltd, UK) and 1.3 μL of first round PCR template. PCR conditions were the same as before but only eight cycles were used. Barcode sequences are shown in Additional file 1: Table S3.

The cleaned DNA was then gel-purified by using the QIAquick Gel Extraction Kit (Qiagen, Valencia, CA, USA) and quantified by using Qubit® (Thermo Fisher Scientific, USA). PCR products were pooled together at a concentration of 10 nM and paired-end sequencing was carried out using a NovaSeq 6000 system.

### Bioinformatic pipeline

Sequence analysis was performed with our bioinformatic pipeline as described previously [49–51].Firstly, quality filtering and trimming (> Q33 Phred quality score) was performed on all the reads of the 16 s rRNA V1 hypervariable region using the Sickle (v.1.2) software [78]. Read error correction was carried out by using the BayesHammer module within the SPAdes (v.2.5.0) software to obtain high-quality assemblies [79]. Paired-end reads were merged (overlap length 50 bp) by using PANDAseq (v.2.11) with the simple Bayesian read merging algorithm [80, 81]. Thereafter, merged reads were dereplicated, sorted, and chimaeras and singletons were removed by using VSEARCH (v.2.3.4; [82]). Sequences were decontaminated against the *Salmo salar* genome using DeconSeq (v.0.4.3; [83]) and overlapped reads were clustered into operational taxonomic units (OTUs) using VSEARCH at 97% sequence identity. OTUs were taxonomically classified against the SILVA 132 database [84] and annotated using the Scikit-learn algorithm implemented in QIIME2 [85, 86].

### Post OTU statistics

All data were analysed in R [68] using the microeco package [87, 88]. After uploading the required files OTUs which were not assigned to the kingdoms of bacteria and archaea were removed. In addition, taxa classified as chloroplast or mitochondria were considered as contamination and discarded. Samples were rarefied to 10,000 reads to limit the number of effects on diversity measurements. Samples of the gut compartments PC and MG were analysed separately. The significance of alpha diversity indices was assessed using Kruskal–Wallis (KW) rank sum test in combination with pairwise Wilcoxon for multi-level factors. In addition, we used a linear model to display significant variables. Beta diversity was measured using weighted UniFrac distances [89]. To identify significant differences among grouping variables (e.g., genetic origin, AGD severity), permutational multivariate analysis of variance (PERMANOVA) was performed based on the weighted unifrac dissimilarity matrix. Fast expectation–maximization for microbial source tracking (FEAST) [90] was used to assess the contribution and the relative importance of fish feed and water bacteria to intestinal microbial communities of starved and fed fish. Thereby, the microbial communities of individual fish served as sink and feed and water samples (both collected at the same sampling timepoint) served as source. Differential abundance was carried out to obtain important indicator taxa by using random forest analysis [91–93]. MeanDecreaseGini was used to determine the importance of differentially expressed taxa. *P* values were adjusted for multiple comparisons using the Benjamini–Hochberg method [94].

The experiments and measurements carried out in this study were conducted under licence (AE19130-P056) of the Health Products Regulatory Authority (HPRA) of Ireland.

## Results

### Health trajectories: wild fish show the highest mortality

**Fig. 2:**
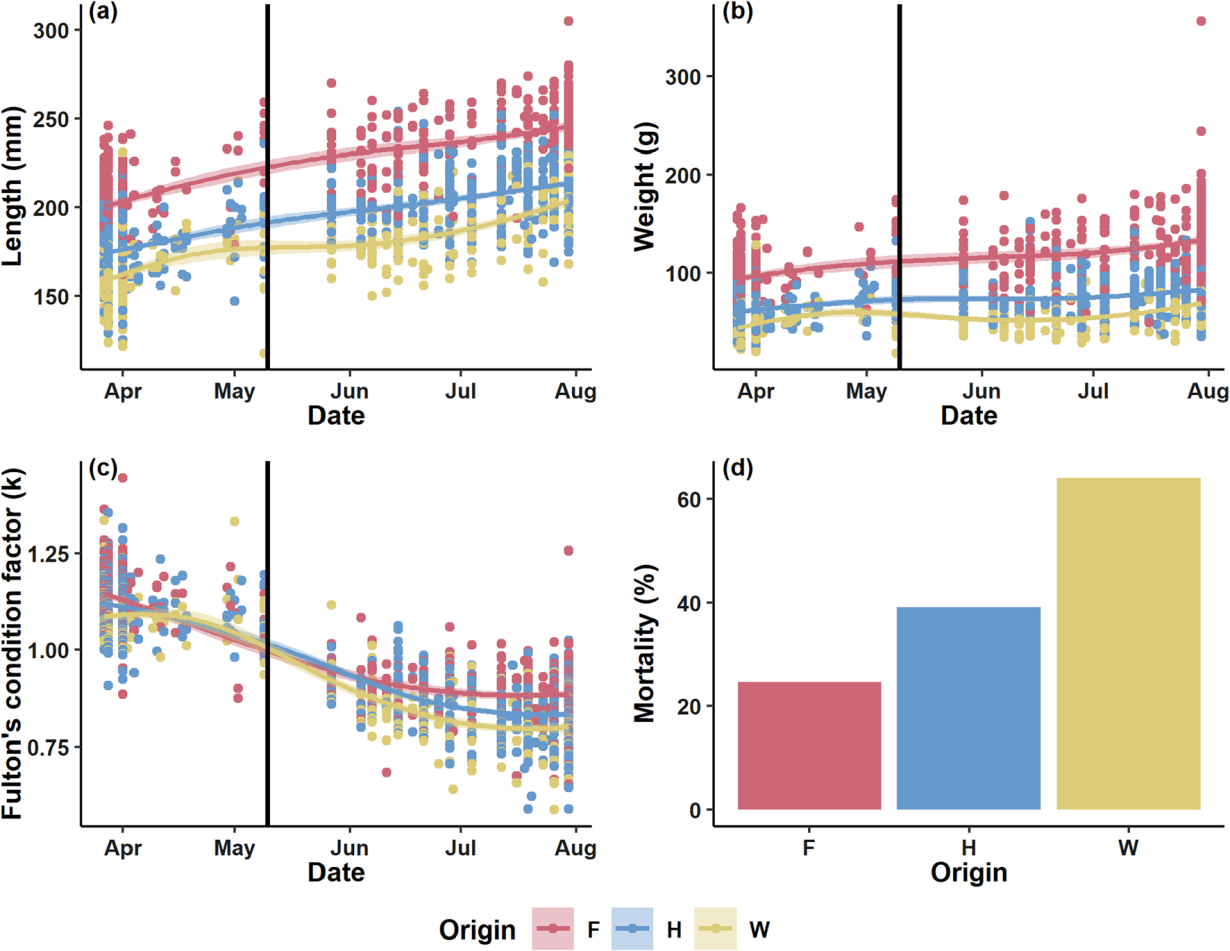
Loess regression of growth trajectories (length (**a**), weight (**b**) and Fulton’s condition factor (**c**)) of fish from different genetic backgrounds throughout the experiment. The black line indicates the point in time of the transfer from the freshwater stage to the sea pens. Mortality (**d**) was calculated as the difference in fish counts between the start and end of the experiment. Origin: F = Farmed, H = Hybrid, W = Wild

From the end of March to August 2019 length and weight measurements for 1,949 fish were obtained. For this analysis the two hybrid groups were combined. The farmed fish were bigger than the other two groups (Fig. 2; Additional file 1: Table S4). Fish grew most during ad libitum feeding in the hatchery. After sea site transfer farmed fish gained on average 11 cm in length, whilst maintaining the same weight. Hybrid fish increased by 23 cm in length and 7 g in weight, whilst wild fish increased in length by 31 cm and 14 g in weight (Additional file 1: Table S4). During the ad libitum feeding in the hatchery phase all fish had a Fulton’s condition factor (k) of above 1, regardless of their origin. After the sea site transfer the condition factor declined for all groups, with farmed fish (K(F) = 0.9) having on average a higher condition than hybrid or wild fish (K(H) = 0.85; K(W) = 0.82; Additional file 1). Farmed fish had on average significantly lower the lowest % water content (74.5 ± 2.7%), followed by the two hybrid groups (HFF: 76.1 ± 1.8%, HWF: 76.8 ± 2.0%) and the progeny of wild fish (77.3 ± 2.32; Additional file 2: Fig. S1.

Mortality was calculated as the difference in fish counts between the start and the end of the experiment for the mixed (4th) sentinel pen. Here, wild fish showed by far the highest mortality rate (64.1%), followed by the hybrid group (39.2%) and farmed fish (24.6%) (Fig. 2).

### Metabolic rate: AGD severity leads to a decline in oxygen consumption

**Fig. 3:**
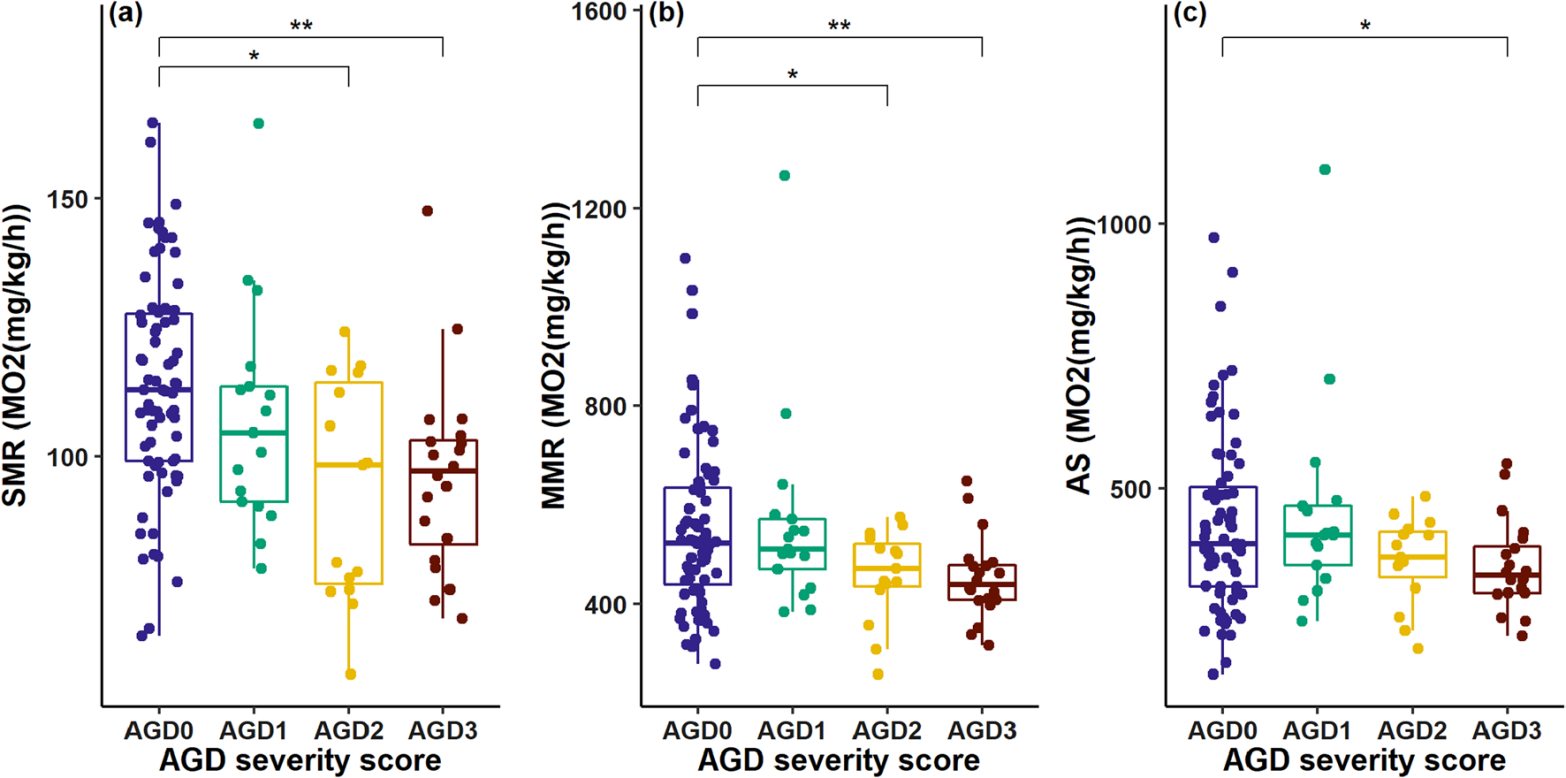
Calculated oxygen consumption (MO_2_(mg/kg/h)) for fish affected by AGD at different severity levels. No differentiation between genetic background was made. Standard metabolic rate (**a**), maximum metabolic rate (**b**) and aerobic scope (**c**). Significance was determined by pairwise *t*-testing against weight adjusted metabolic rate measures of fish with an AGD score of 0. *P*-values adjusted by Bonferroni correction. Significance codes are only shown for significant results: **p < 0.01; *p < 0.05

The metabolic rates of post-smolt Atlantic salmon were determined over 6 weeks. 55 fish showed signs of AGD with their AGD severity scores ranging from very light (score 1: 17 fish) to moderate (score 3: 23 fish). Signs of AGD were mostly observed towards the end of the experiment. Overall, the fish’s origin had no significant impact on neither SMR (*p* = 0.25), MMR (*p* = 0.51) or AS (*p* = 0.43). Simple pairwise testing of weight-adjusted metabolic rate measures revealed a significant decline of such with increasing AGD severity (SMR: AGD0 vs. AGD 2: *p* = 0.01; AGD0 vs. AGD3: *p* = 0.004; MMR: AGD0 vs. AGD 2: *p* = 0.02; AGD0 vs. AGD3: *p* = 0.005; AS: AGD0 vs. AGD3: *p* = 0.03; Fig. 3). We chose to confirm pairwise testing results by an ANCOVA (Anova Type III), using weight as a covariate. The model revealed significant differences in SMR and MMR means between fish with different AGD severity scores (SMR: F = 7.38, *p* = 0.0001; MMR: F = 3.79, *p* = 0.01). However, in contrast to the formerly mentioned pairwise *t*-testing no significant differences in AS were observed (AS: F = 2.15, *p* = 0.09; Additional file 1: Table S5). Multiple comparisons of means (Tukey post-hoc) revealed significantly lower metabolic rate measures between AGD 0 versus AGD 2 and 3, respectively (SMR: *p*(0/2) = 0.002, *p*(0/3) = 0.002; MMR: *p*(0/3) = 0.03) (Additional file 1: Table S6). Important to note that the observable dispersion of metabolic rate measurements for fish with an AGD score of 0 (SD(SMR = 21.22; MMR = 168.07)) is larger than for fish with AGD scores of 3 (SD(SMR = 18.52; MMR = 83.49); Fig. 3).

### The gut microbiome of starved and fed fish: feeding status matters

**Fig. 4:**
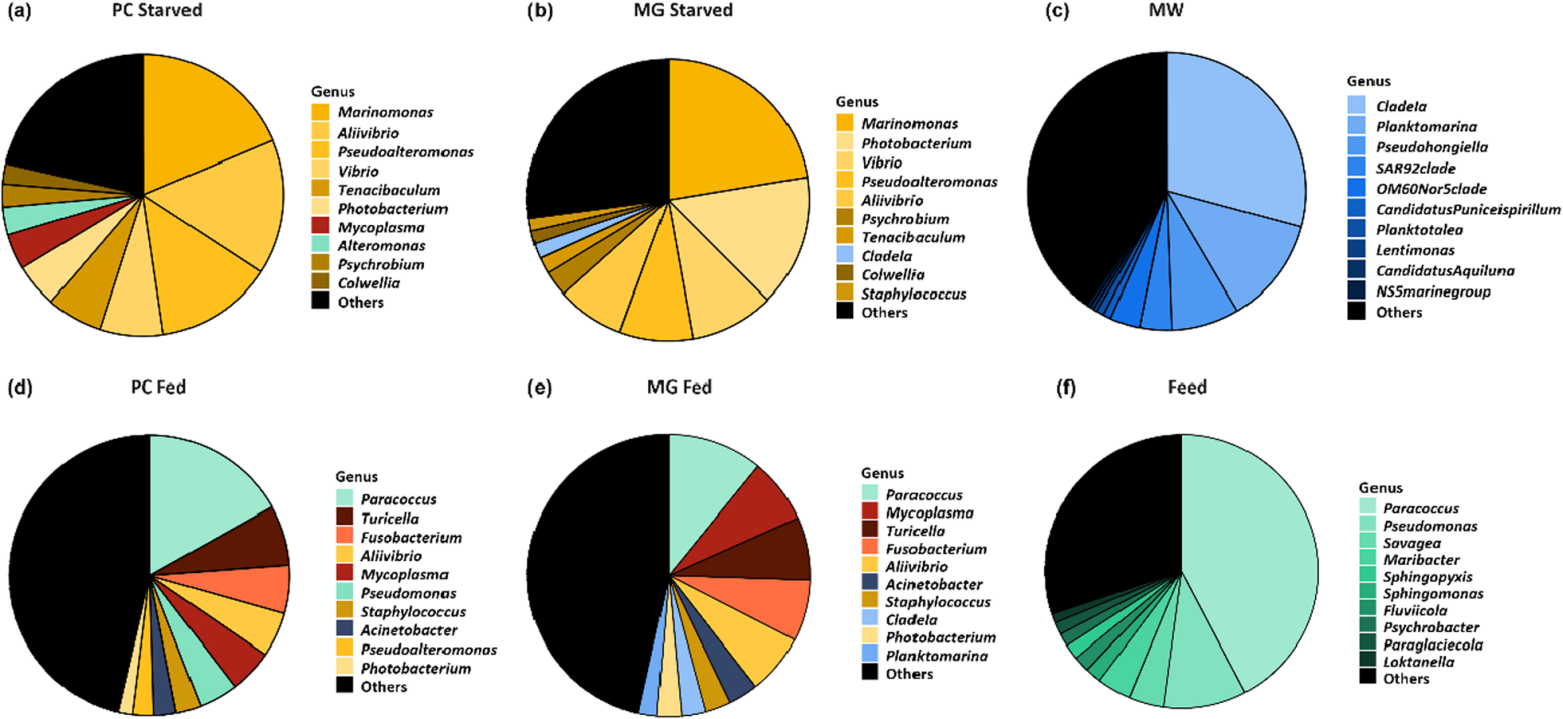
Pie charts showing the mean relative abundance of the 10 most abundant taxa on genus level for pyloric caeca of starved fish (**a**), midgut of starved fish (**b**), marine water (MW (**c**)), pyloric caeca of recently fed fish (**d**), midgut of recently fed fish (**e**) and fish feed (**f**). Starved fish were without food for at least 48 h. For illustration purposes samples of T0 and T1 were pooled together

**Fig. 5:**
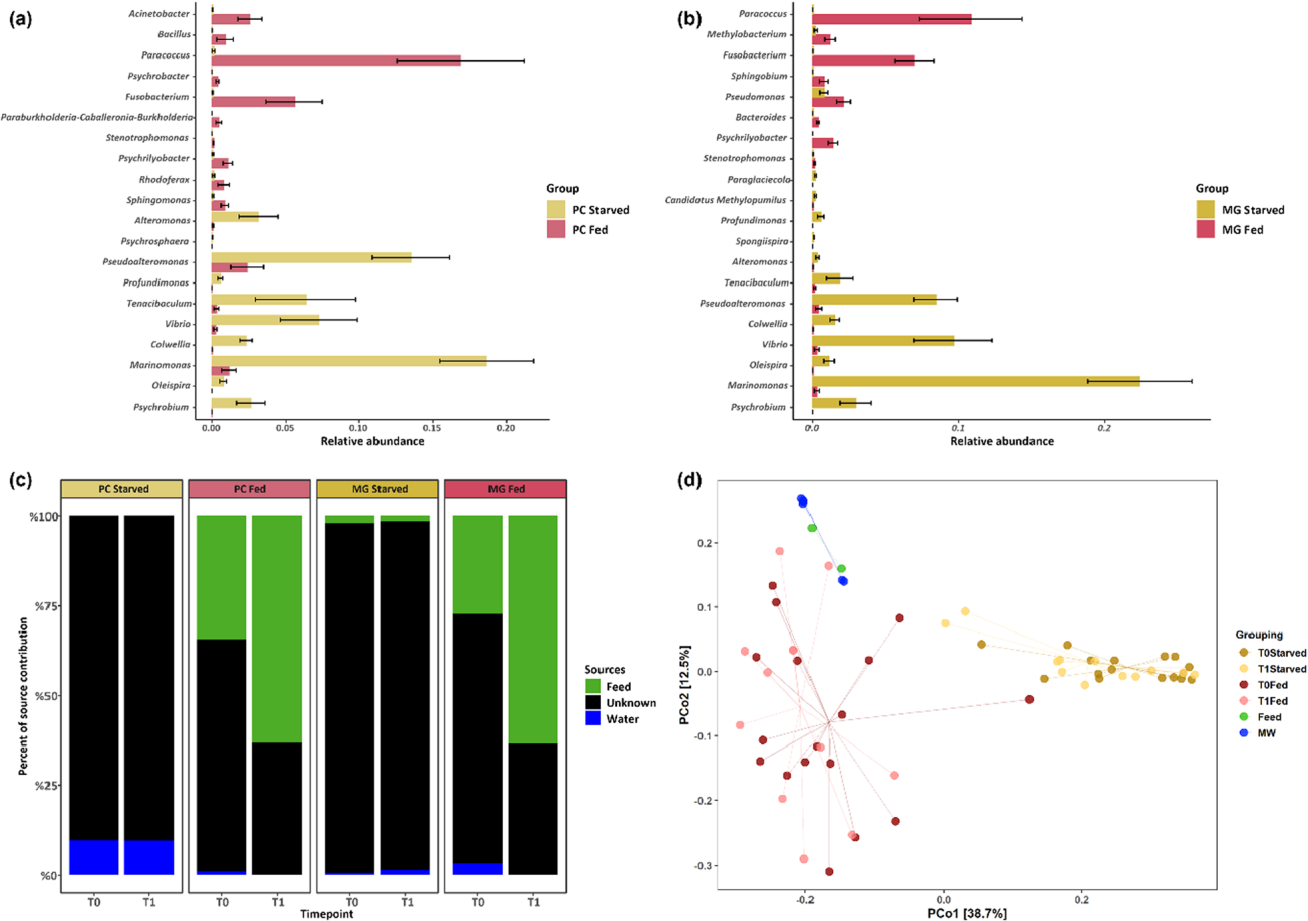
Differential abundance analysis between starved and fed fish in PC samples (**a**) and MG samples (**b**). Important genera were determined with random forest classification. Mean decrease gini served as indicator value. **c** Shows the source tracking analysis determined by the FEAST framework. Bars show the average contribution of each source (either feed, water or unknown) to the overall microbial community for different gut compartments, feeding status and timepoints. **d** Illustrates a principal coordinates analysis (PCoA) derived from weighted UniFrac distances between environmental samples (MW = Marine water and feed), starved fish ((48 h feed withdrawal) and recently fed fish at two different points in time (T0 and T1). T1 was sampled 14 days after T0. Lines mark the position of the centroids of each group. For illustration purposes no differentiation between PC and MG samples was made. However, differentiation was included in Permanova calculations (Additional file 1: Tables S7, S8)

In an auxiliary experiment, we compared recently fed and starved fish at two different timepoints (14 days apart). The results in this paragraph are based on samples from this side experiment. At a phylum level, gut microbial communities were mostly dominated by *Proteobacteria*. However, there was an observable difference in relative abundance between fed and starved fish. In fed fish the mean relative abundance of *Actinobacteria* (Fed: PC 14.6%, MG 13.8%; Starved: PC 1.3%, MG 4.3%), *Firmicutes* (Fed: PC 8.7%, MG 8.4%; Starved: PC 0.7%, MG 2.0%) and *Fusobacteria* (Fed: PC 6.7%, MG 8.4%; Starved: PC 0.09%, MG 0.01%) were higher than in starved fish, whereas *Proteobacteria* (Fed: PC 57.5%, MG 54.3%; Starved: PC 83.4%, MG 86.5%) were less abundant (Additional file 2: Fig. S2). At genus level, starved fish guts were dominated by *Marinomonas*, *Vibrio*, *Photobacterium*, *Aliivibrio*, *Pseudoalteromonas* and *Tenacibaculum* (Fig. 4a, b). The top 10 most relative abundant genera accounted for app. 75% of total relative abundance in both PC and MG samples. In recently fed fish, the top 10 genera accounted for app. 55% of total relative abundance. Here, most abundant taxa were *Paracoccus*, *Turicella*, *Mycoplasma*, *Fusobacterium* and *Aliivribio* (Fig. 4d, e). Feed samples were mostly dominated by *Paracoccus* and *Pseudomonas* and water samples (MW) were dominated by genera *Clade Ia*, *Planktomarina* and *Pseudohongiella* (Fig. 4c, f). Differential abundance analysis revealed that in PC samples 69 taxa were significantly different between fed and starved fish and 31 taxa in MG samples. The most striking differences between starved and fed fish were observed for *Marinomonas* (Fed: PC 1.2%, MG 0.3%; Starved: PC 18.6%, MG 22.3%), *Pseudoalteromonas* (Fed: PC 2.4%, MG 2.8%; Starved: PC 13.5%, MG 8.4%), *Vibrio* (Fed: PC 0.2%, MG 0.2%; Starved: PC 7.2%, MG 9.6%), *Acinetobacter* (Fed: PC 2.6%, MG 3.4%; Starved: PC 0.03%, MG 0.9%), *Fusobacterium* (Fed: PC 5.6%, MG 7.0%; Starved: PC 0.04%, MG 0.01%) and *Paracoccus* (Fed: PC 16.8%, MG 10.9%; Starved: PC 0.1%, MG 0.01%; Fig. 5a, b). We used source tracking analysis to determine the overall contribution of feed and water bacteria to fed and starved fish guts. In fed fish around 50% of observed bacteria originated from feed samples, whereas water bacteria were only a minor source for the gut microbial community composition (Fig. 5c). PCoA analysis separated starved, fed and environmental samples, confirming differences in community composition between the groups (Fig. 5d). Within groups, recently fed fish showed significant differences between timepoints T0 and T1 in MG samples (F = 2.2, R^2^ = 0.14, *p* = 0.03) but not in PC samples (F = 1.8, R^2^ = 0.14, *p* = 0.09). There was also no significant difference within the two timepoints of starved fish, regardless of the sampled gut compartment (PC: F = 0.93, R^2^ = 0.07, *p* = 0.48; MG: F = 1.3, R^2^ = 0.09, *p* = 0.27; Additional file 1: Tables S7, S8).

### Alpha diversity: lower microbial richness in AGD infected fish

**Fig. 6:**
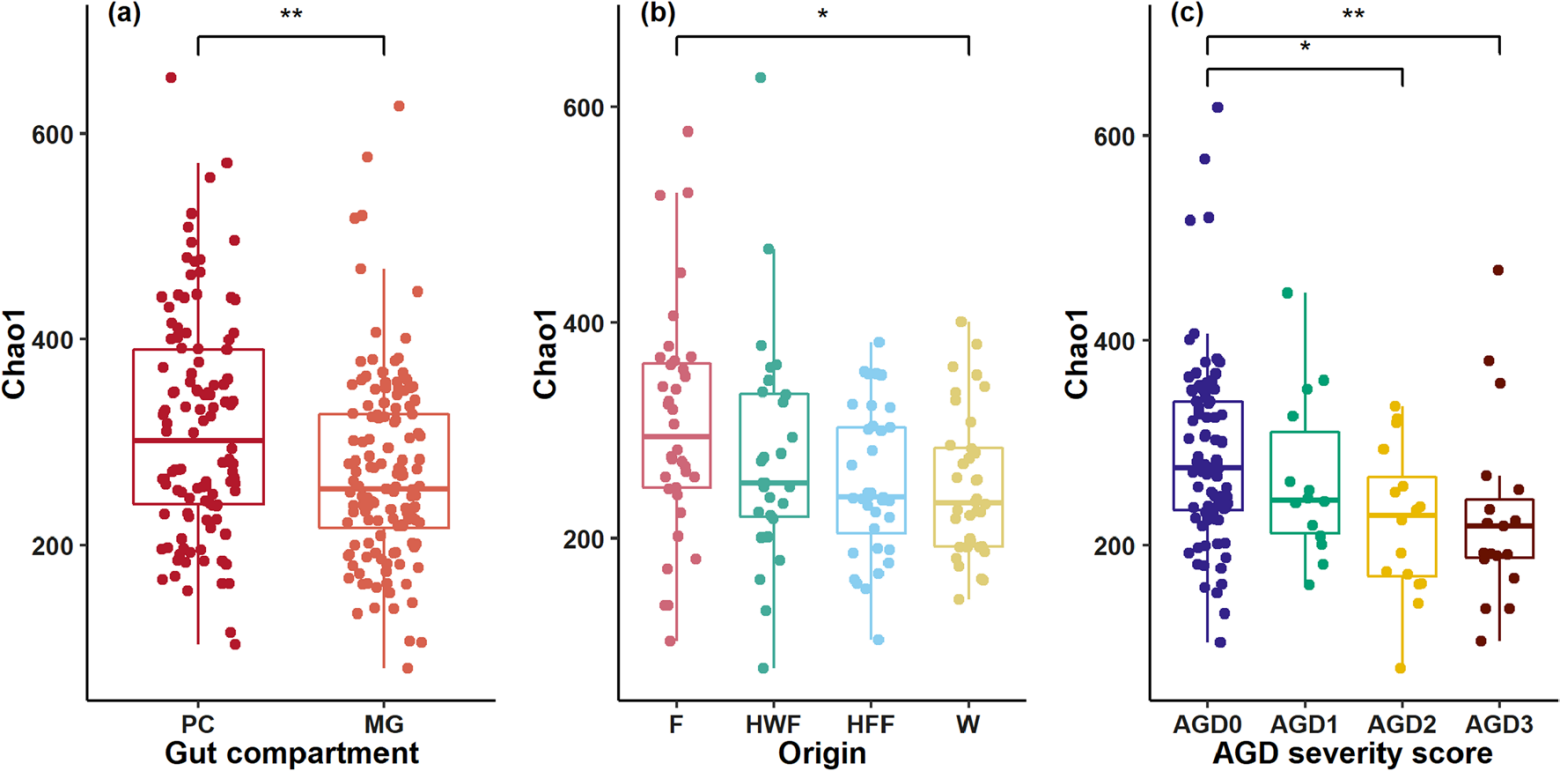
Chao1 richness estimates for multiple comparisons for starved fish between gut compartments (**a**), genetic background in MG samples (**b**) and AGD severity in MG samples (**c**). Lower microbial richness in MG samples compared to PC samples. In MG samples wild fish show a lower microbial richness than farmed fish. Fish with an AGD severity score of 2 and 3 showed a lower microbial richness than non-symptomatic fish (AGD 0). PC = Pyloric caeca, MG = Midgut, F = Farmed, HWF = Hybrid Wild Female, HFF = Hybrid Farmed Female, W = Wild. Significance was determined by Dunn’s test for multiple comparisons of groups of significant Kruskal–Wallis results. Significance codes shown for significant differences: **p < 0.01; *p < 0.05

All following analysis refer to samples from the metabolic rate experiment. PC samples showed a significantly higher alpha diversity than MG samples (Chao1: KW, *p* = 0.001; Shannon: KW, *p* = 0.001; Fig. 6a). In PC samples pairwise testing revealed no significant impact of genetic origin on diversity measures. However, Shannon and InvSimpson indices showed a significantly lower diversity for the samples with the highest AGD score 3 compared to AGD score 0 (InvSimpson: KW, Z = 3.44, *p* = 0.003) and AGD score 1 (Shannon: KW, Z = 2.91, *p* = 0.02; InvSimpson: KW, Z = 2.92, *p* = 0.01). In MG samples several diversity indices showed significantly lower values for samples with AGD score 3 than AGD score of 0 (Chao1: KW, Z = 3.20, *p* = 0.008; Shannon: KW, Z = 3.00, *p* = 0.01; InvSimpson: KW, Z = 3.05, *p* = 0.01; Fig. 6c). In addition, Chao1 richness estimates were lower in fish of wild origin than in farmed (Chao1: KW, Z = 2.69, *p* = 0.04; Fig. 6b). To investigate this relationship further we tested the combination of influencing factors by fitting linear models. The model results for the interaction term of AGD severity score and genetic origin on Chao1 richness estimates are displayed in Additional file 1: Table S9. Fish with an AGD score of 3 showed a significantly lower richness than fish with a score of 0 (t =  − 3.1, *p* = 0.002). Fish from HFF and wild origin had significantly lower richness compared to farmed fish (HFF: t =  − 2.39, *p* = 0.01; W: t =  − 2.29, *p* = 0.02). When affected by AGD (AGD score = 3) our model detected a significant lower microbial richness in farmed fish compared to wild (t = 2.08, *p* = 0.04) and hybrid farmed fish (t = 2.70, *p* = 0.007). However, due to the low number of observations (n = 3) per origin for an AGD score of 3 these interaction results must be treated with care.

### Beta diversity: community shift in AGD infected fish

**Fig. 7:**
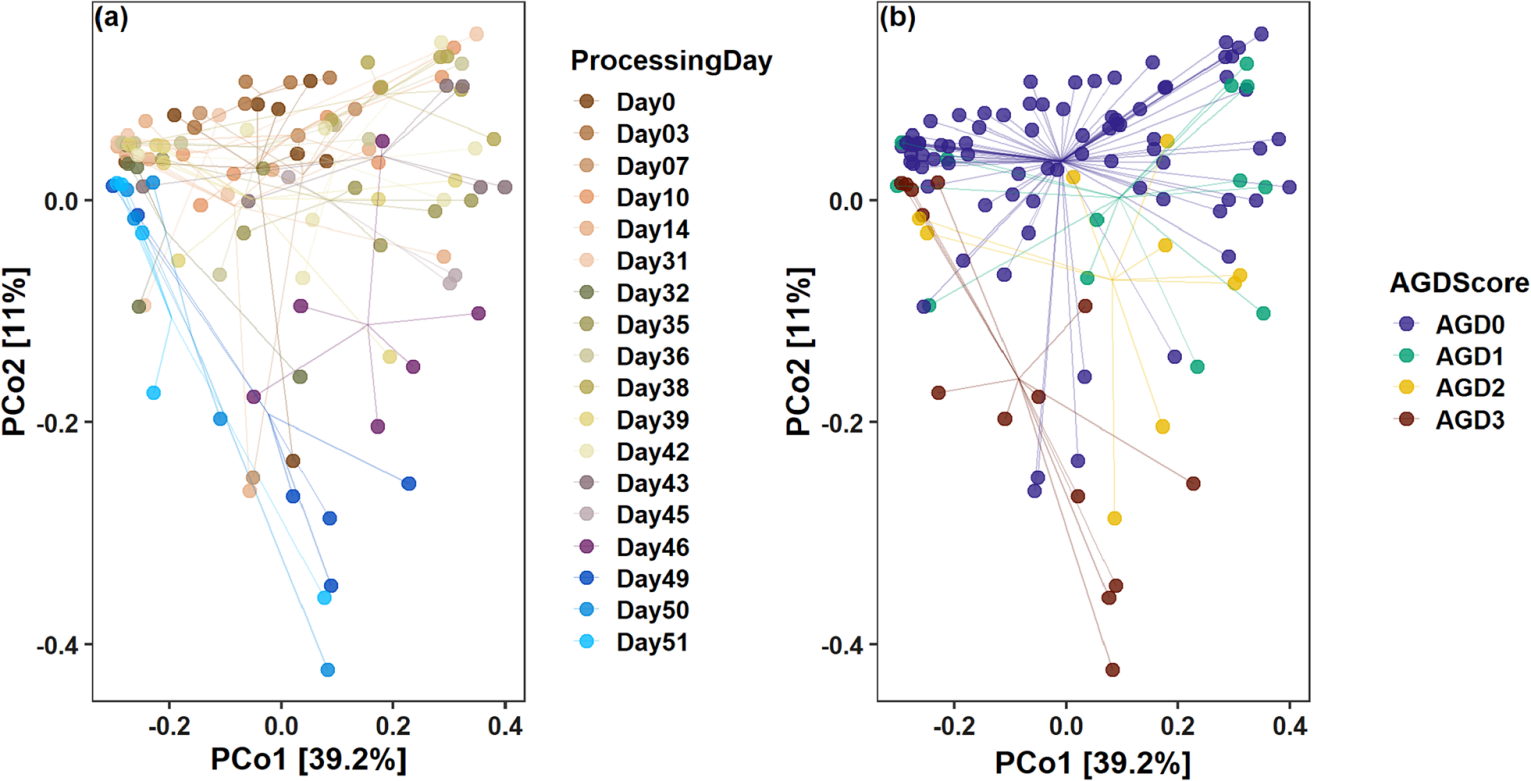
Principal coordinates analysis (PCoA) derived from weighted UniFrac distances among PC samples from starved fish. Samples are coloured according to the day when fish were dissected (**a**). Thereby, Day 0 marks the start of the respirometry experiment. **b** shows the same distribution, but here samples are coloured according to the associated AGD severity level. Connected lines mark the position of the different group centroids

PCoA ordination separated microbial communities of PC samples by processing days and AGD severity score along PCoA1, which captured 39.2% of the variation, whilst most fish associated with an AGD score of 3 were separated by PCoA2 (Fig. 7). Whilst samples at the earlier stages of the experiment seem to cluster together randomly, samples taken at the later stages of the experiment, especially from Day 46 onwards seem to have a higher likelihood to have a different community structure. Most of those samples had a higher AGD severity score, which implies an effect of symptomatic AGD infections on gut microbial communities. To confirm our observations, we tested our variables of interest by applying Permanova sequentially. Processing day (dissection day of the fish) explained 25.2% of variation (F = 2.2, R^2^ = 0.25, *p* = 0.0001), AGD severity score was also significant and explained 9% of variation (F = 4.6, R^2^ = 0.09, *p* = 0.0001), but there were no significant differences regarding genetic origin (F = 1.32, R^2^ = 0.02, *p* = 0.16). 62.6% of variation remained unexplained (Additional file 1: Table S10). To test for differences within the two significant factors of interest we conducted groupwise comparisons. We observed significant differences in community composition between samples with an associated AGD score of 0 and other AGD severity groups. In addition, samples with an AGD score of 1 differed significantly from samples with an AGD score of 3, whilst there were no significant differences between groupings of AGD score 1 and 2, nor between AGD score 2 and 3 (Table 2). Permutation test for homogeneity of multivariate dispersion revealed that the dispersion between AGD groups did not differ (F = 0.97, *p* = 0.411). As for day-by-day comparisons, samples taken at the beginning of the study do show similarities with each other and differ significantly from samples of later stages (Additional file 1: Table S12), which matches the difference in AGD scores and a gradual rise in temperature (R^2^ = 0.99). However, there are several “outlier” days where community compositions do not differ significantly e.g., the community composition from processing days 36 and 39 are not significantly different from the community composition of samples on day 0. Similar patterns were observed for samples derived from fish midguts. Results are displayed in Additional file 1: Tables S12 and S13.

**Table 2:** Permutational analysis of variance (PERMANOVA) testing pairwise comparisons for pyloric caeca (PC) samples from different AGD severity groups

Groups	F	R^2^	*p*.value	*p*.adjusted	Significance
AGD0 versus AGD1	2.97640	0.03167	0.017	0.025	*
AGD0 versus AGD2	3.91483	0.04306	0.002	0.006	**
AGD0 versus AGD3	6.17319	0.06288	0.001	0.006	**
AGD1 versus AGD2	1.50253	0.06987	0.161	0.161	
AGD1 versus AGD3	3.43869	0.12091	0.012	0.024	*
AGD2 versus AGD3	1.96371	0.08551	0.078	0.093	

Distance matrix calculated by weighted UniFrac measure. Permutations used: 9999. Significance codes: **p < 0.01; *p < 0.05

### Differential abundance

**Fig. 8:**
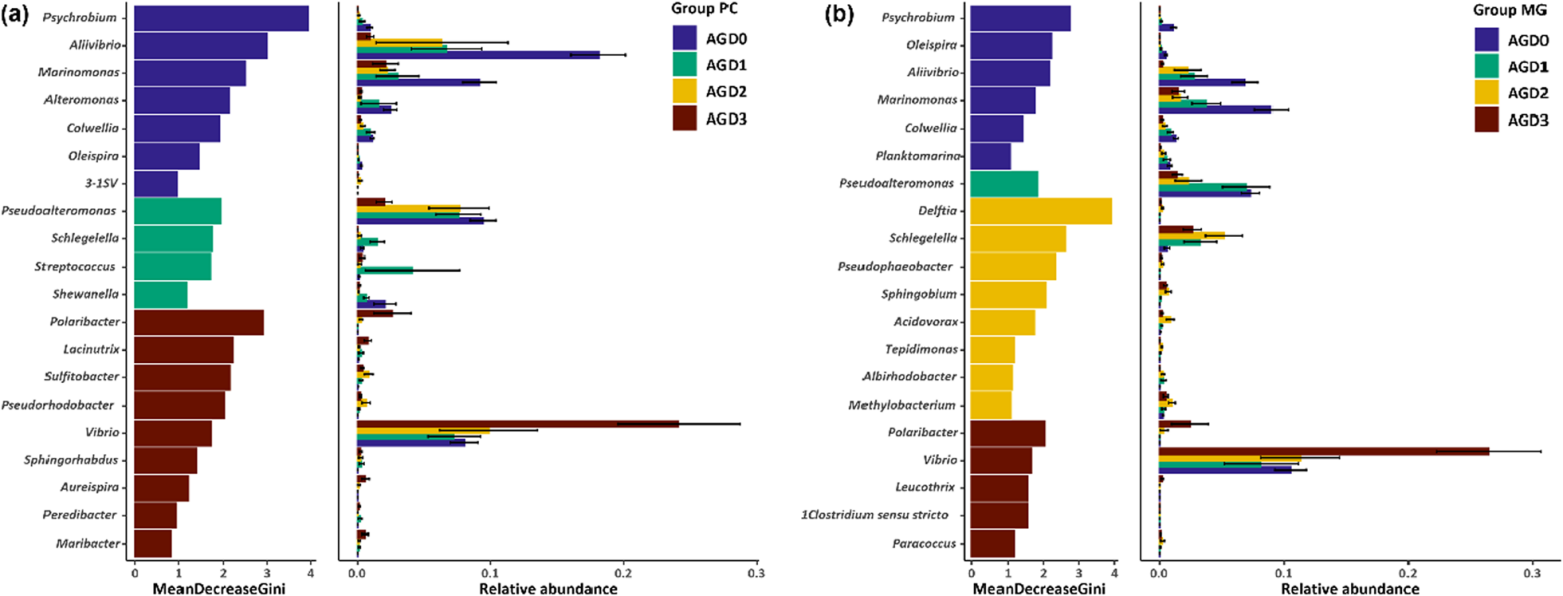
Top 20 differentially abundant genera in PC samples (**a**) and MG samples (**b**) in starved fish determined by random forest analysis. Mean Decrease Gini indicator represents the importance of each genus in distinguishing between AGD severity scores. Right side of each graph depicts the relative abundance of genera per AGD severity group. Error bars indicate standard deviation.

We found for both PC and MG samples 45 taxa which differed significantly between the four AGD severity stages. *Psychrobium* showed the highest measure of importance in differential abundance for AGD severity score (Fig. 8a, b). However, overall *Psychrobium* only shows a low relative abundance. In PC samples, *Aliivibrio*, *Marinomonas* and *Pseudoalteromonas* show a lower abundance in fish with an AGD score of 3 compared to AGD 0, whereas *Polaribacter* and *Vibrio* show a higher abundance in symptomatic fish (Fig. 8a). *Photobacterium*, the genus with the overall highest mean relative abundance of app. 25% in PC samples wasn’t significantly differentially abundant (data not shown). In general, we observed similar trends in taxa abundance changes for both PC and MG.

## Discussion

All fish, regardless of genetic origin, showed a decline in Fulton’s condition factor after transfer to the sea pens. A common observation since fish will often lose appetite on transfer from fresh to seawater [62]. In addition, changes in food availability (ad libitum vs. maintenance feeding) and rising water temperatures accompanied by emerging AGD infections might have contributed to this reduction in condition. Surprisingly, wild fish gained on average more weight and length than hybrid and farmed fish after the sea pen transfer. However, wild fish also showed higher mortality. Size-selective mortality may explain the relative increase in size. Body size appears to have impacted the survival of the progeny of the farmed group to a lesser degree. There are several possible explanations for this observation. Firstly, farmed fish are genetically improved to maximize growth and are therefore bigger and heavier than their wild and hybrid counterparts [14]. In our study farmed fish showed on average the lowest percentage of water content. Since a low water content is negatively correlated with fat content [74], one might hypothesise that farmed fish had more fat reserves and therefore potentially a higher chance of survival under our experimental conditions. In addition, farmed fish might have developed different coping mechanisms for stressors compared to wild and hybrid fish (reviewed by [95]), eventually leading to the observed differences in cohort mortalities.

Towards the end of the experiment fish had clear signs of AGD regardless of their genetic background. Fish with more severe AGD infections had on average a significantly lower standard metabolic rate and maximum metabolic rate compared to the non-symptomatic fish at the beginning of the experiment. ANCOVA revealed no significant differences in AS. At first glance this might be a surprising find. However, AS is calculated as the difference of MMR and SMR. Hence, if SMR and MMR “decline” at a similar rate, the AS value will still stay roughly consistent between groups. Respirometry uses oxygen consumption as a proxy for host metabolic rate [96]. AGD infections likely lead to a decrease in oxygen absorption capacity of fish gills, resulting in lower metabolic rate measurements. Hvas et al. [37] showed that AGD infected Atlantic salmon had significantly lower MMR and AS compared to healthy fish. They concluded that changes occurring in the gill because of an infection with *N. perurans* can lead to compromised gas exchange and ion regulation across the gills, potentially affecting appetite, growth and overall survival [37]. This finding corresponds with our findings of lower MMR values in AGD infected fish. However, we also noted a reduction in SMR. Previous studies have not detected any differences in SMR either between healthy and sick fish with advanced signs of AGD (AGD Score of 4 and above) [37] or between different fish species [97, 98]. In addition to the significant decline in SMR, we also observed a greater dispersion of metabolic rate measures in fish with no signs of infection. For symptomatic fish this dispersion declines. Individuals with high metabolic rates were rarely observed in symptomatic fish. AGD infections might have led to higher mortalities in fish with high SMR and/or MMR during the experiment, resulting in selection for fish with comparatively lower metabolic rates. Previous research has shown that a higher SMR can be beneficial when resources are plentiful, but detrimental should environmental conditions deteriorate [32]. This phenomenon is also consistent with the “allocation hypothesis” described in other animals. The allocation hypothesis suggests a fitness advantage for lower standard metabolic rates because of lower maintenance costs, which allows for the reallocation of energy towards growth and increased immune function [99–102]. Consequently, low oxygen intakes induced by AGD might lead to an earlier state of hypoxia and eventually death in fish with high metabolic rates compared to fish with a lower maintenance cost. To establish the true metabolic status of AGD-infected fish and the validity of the allocation hypothesis, it may be valuable to establish more direct measures of metabolic rate, for example via measures of muscular mitochondrial efficiency and ATP production [103, 104]. Such direct measures would establish whether AGD truly selects for fish with lower metabolic demands, rather than simply inducing a state of chronic hypoxia.

Metabolic rate differences among fish from different genetic backgrounds were not observed. Fish with a higher SMR have been shown to harvest energy from ingested food more rapidly, which could be reflected in a greater growth potential [35]. By this logic, we hypothesized that farmed fish should have a higher SMR than wild fish. In addition, in a common garden experiment [105] found higher AS among the progeny of wild fish from the Burrishoole catchment than the offspring of farmed fish, potentially due to differences in life history traits [105]. It is possible that within the context of our common garden experiment, uniform environmental conditions counterbalanced potential genetic differences in metabolic phenotypes. Therefore, we assume a strong impact of the environment on a fish’s metabolic profile. However, genetic effects might still be very important in the wild, especially in the context of evaluating fitness differences between wild and hybrid populations.

To accurately estimate metabolic rates fish were starved before going into respirometry. Whilst it is commonly accepted that diet can affect microbial community composition [106, 107] the prior feeding status of a fish is hardly considered when investigating gut microbial communities [108]. However, due to the process of feeding and the consequent introduction of associated bacteria, an individual’s observable microbiome at the time of sampling might be a carryover from the individual’s last meal [109]. The consequential variation makes a precise discrimination between drivers of community composition and noise extremely difficult. We found significant differences in gut microbial community composition between fed and starved fish. In fed fish three of the top ten most abundant taxa likely originated from the environment. *Paracoccus* and *Pseudomonas* from feed samples and *Clade Ia* from the surrounding water. Since those taxa were not abundant in starved fish, we can assume they are allochthonous in origin. By comparing gut microbial community composition at two points in time 14 days apart we found in MG samples significant differences between fed fish but not between starved fish. It so seems that the presence of food increases the relative abundance of certain taxa (e.g., *Fusobacteria*, *Psychryliobacter* and *Acinetobacter*), whereas the relative abundance of taxa, which are dominant in starved fish decreases (e.g. *Marinomonas*, *Vibrio* and *Pseudoalteromonas*). It is possible that the presence of food changes the nutritional niche within the gut, thereby favouring taxa which metabolise the introduced nutrients. In times of food sparsity those taxa are outcompeted by bacteria found in starved fish. We conclude that in our experiment starved fish harbour mostly autochthonous bacteria and an investigation of changes in community composition will not be biased by fish feeding status. In addition, since there was no difference between gut microbial community composition in starved fish between T0 and T1 for neither gut compartment, short periods of food withdrawal do not seem to impose changes on overall gut microbial community composition. However, an observation over longer time periods is warranted to draw definite conclusions.

We used a common garden approach undertaken under farm conditions to evaluate the impact of AGD and co-occurring environmental factors on gut microbial community composition. By doing so we kept diet and environmental conditions constant for all fish throughout their life, thereby reducing the number of factors impacting host microbiomes, opening the possibility to estimate the potential influence of host genetics. In our experimental setup a fish’s genetic background played only a minor role in explaining inter-individual differences. In midgut samples, we did find a significantly higher diversity in farmed fish compared to wild and hybrid-farmed-female fish. Interestingly, this finding was reversed when fish were affected by AGD. However, given the high variability between fish, the latter observation must be treated with care as we just had three samples per origin for that category. A big part of the observed variation in beta diversity could not be explained by environmental drivers. An expected result, since each individual has its own gut microbial ecosystem, which is impacted by its very own intrinsic dynamics [110]. Thereby, an individual’s “ecosystem” is not only shaped by environmentally induced selective pressures but also by stochastic processes, like dispersal (e.g., the exchange of microbes with the environment) or drift (the natural occurrence of death, reproduction and replacement [48, 49, 111]. Nevertheless, Permanova testing also revealed that community composition differed by time (processing/sampling day) and a fish’s AGD severity score, indicating a role of environmental factors to influence gut bacteria profiles. Rising water temperatures throughout the experiment might be a possible explanation for the time component. In ectotherms temperature is known to play a major role in shaping gut microbial communities [112–114]. All bacteria have optimal growing temperatures determined by thermodynamic limitations [115]. It is therefore plausible that some taxa were affected by the temperature increase, consequently leading to a change in abundance due to changes in their relative ecological fitness. AGD severity score was also highly significant in explaining inter-individual differences in community composition. In fish with an AGD severity score of 3 we observed significantly less microbial richness and evenness compared to non-symptomatic fish. This implies a decline in the overall number of taxa as well as a dominating effect resulting in fewer highly abundant taxa. In addition, beta diversity analysis showed a shift in taxa abundance between symptomatic and non-symptomatic fish. Differential abundance analysis in combination with correlation analysis showed abundance changes for several taxa among disease states. Thereby we observed a gradual decline in highly abundant taxa like *Aliivibrio*, *Marinomonas* and *Pseudoalteromonas*, whereas *Vibrio* increased in symptomatic fish. Perturbation of microbial communities due to fish disease has been described in several studies [57, 116–118]. In homeostatic conditions commensal bacteria can resist colonization or growth of potentially pathogenic taxa by occupying all available ecological niches [119]. However, if conditions change e.g., due to host stress or infection, the microbial barrier functions might be disturbed [57, 116]. The degree of community perturbation might be linked to the host’s stress tolerance e.g., maintaining appetite and immune function. It is known that communities showing reduced diversity are less likely of having a species with an opposing trait towards an invader or pathogen [120, 121]. Hence, we predict that a decline in diversity might have a negative impact on the ability of bacterial communities to prevent secondary infections. In addition, the replacement of taxa by few and highly abundant bacteria might result in a reduced capacity to digest a diverse diet, which could negatively impact fish growth and wellbeing [122]. A question very relevant to aquaculture research would be to test if a perturbation of microbial communities is reversible when host health improves (e.g., due to freshwater treatment against AGD infections) and to determine the long-term effects on fish growth. Finally, in the context of a warming future AGD infections might become an even more severe threat for captive and wild populations alike. Due to the inverse relationship between water temperature and water oxygen levels, fish hypoxia and stress are likely to increase. Coupled with an infectious disease like AGD implications for farmed and wild fish might be devastating.

## Conclusion

This study provides a novel and multi-approach exploration of AGD infection on Atlantic salmon physiology. The common garden suggests a strong additive genetic component in AGD susceptibility with the progeny of wild fish, likely to be historically naive in respect of AGD exposure, having intermediate and higher mortality relative to the progeny of hybrid or farmed fish. However, we could not determine categorically if vulnerability to AGD infection was a direct function of a genetically determined immunological response or some other factor associated with genetic background. It is just as likely that a fish’s initial condition or size, also traits governed by genetic background, determines its chances of survival. We thereby assume that a combination of stressors, that include the initial saltwater transfer, the sea pen environment and AGD infections, contributed to the higher mortality in the progeny of wild fish. We also show that infection with AGD limits the respiration capacity of fish, likely leading to hypoxia and health deterioration. Due to our findings, we hypothesize that high standard metabolic rates and associated high maintenance costs, negatively impact a fish’s chances of survival when affected by AGD. Future research might address this question by using more direct measures of metabolic rates e.g., via measures of muscular mitochondrial efficiency and ATP production. Future studies of gut microbial communities in fish should consider a fish’s feeding status. We show that feeding promotes inter-individual differences, which might limit the explanatory power of a study depending on its context. Our study also shows an effect of AGD infections on gut microbial balance such that AGD infected fish had fewer but highly abundant taxa. Microbial communities that show a low diversity are less likely to have species with an opposing trait towards pathogens and might have a diminished capability to digest a diverse diet. Hence, a perturbation of gut microbial community composition might have severe implications for fish growth and general wellbeing, threatening farmed fish and wild populations alike. A question very relevant to aquacultural research would be to test if such a dysbiosis is reversible when host health improves (e.g., due to freshwater treatment against AGD infections) and to determine the long-term effects on fish growth.

## Supplementary Information

**Additional file 1: Table S1**. Overview of the metabolic rate experiment. Sample_Id refers to individual fish. Sampling day refers to the day when fish were caught. Processing day refers to the day fish were dissected. Experiment day equals processing days, only in another format. Temperature refers to the water temperature at the sea pens on each sampling day. **Table S2**. First round PCR primers used for NGS library preparation. **Table S3**. Second round PCR primers used for NGS library preparation. **Table S4**. Mean length, weight and Fulton’s condition factor per genetic origin for 3 points in time (pre sea pen hatchery, date of sea pen transfer and at the termination point of the experiment). **Table S5**. ANCOVA results of the effect of AGD severity Score on weight adjusted SMR (left), MMR (middle) and AS (right) measures. Significance codes: *** < 0.001; ** < 0.01; * < 0.05. **Table S6**. Multiple comparisons of means of metabolic rate measures between different AGD severity scores (Tukey post-hoc for weight-adjusted model). Significance codes: ** < 0.01; * < 0.05. **Table S7**. Permanova results showing differences between gut microbial community compositions of starved and fed fish for two different timepoints T0 and T1 in PC samples. Significance codes: ** < 0.01; * < 0.05. **Table S8**. Permanova results showing differences between gut microbial community compositions of starved and fed fish for two different timepoints T0 and T1 in MG samples. Significance codes: ** < 0.01; * < 0.05. **Table S9**. Linear model for Chao1 richness estimates in MG samples calculated by the interaction term of AGD severity score and genetic origin. AGD severity of 0 and farmed origin served as baseline for comparisons. Significance codes: *** < 0.001; ** < 0.01; * < 0.05. **Table S10**. Permutational analysis of variance (PERMANOVA) results testing the effect of AGD severity score, genetic origin and processing day (day of gut dissection) on pyloric caeca (PC) microbial community composition based on weighted unifrac distance matrices. **Table S11**. Permanova of pairwise comparisons of PC samples grouped by day of their processing. Distance matrix calculated by weighted UniFrac measure. Permutations used: 9999. Significance codes: *** < 0.001; ** < 0.01; * < 0.05. **Table S12**. Permutational analysis of variance (PERMANOVA) results testing the effect of AGD severity score, genetic origin and processing day (day of gut dissection) on midgut (MG) microbial community composition based on weighted unifrac distance matrices. **Table S13**. Permutational analysis of variance (PERMANOVA) testing pairwise comparisons for midgut (MG) samples from different AGD severity groups. Distance matrix calculated by weighted UniFrac measure. Permutations used: 9999. Significance codes: **p < 0.01; *p < 0.05.

**Additional file 2: Fig. S1**. Percent water content of fish from different origins during the sea pen experiments. Data derived from fish of mixed (4th) sentinel pen over the course of the experiment. Significance was determined by pairwise *t*-testing against fish from farmed origin. F = Farmed, HFF = Hybrid Farmed Female, HWF = Hybrid Wild Female, W = Wild, Significance codes: ***p < 0.001; **p < 0.01. **Fig. S2**. Stacked bar plot showing the mean relative abundance of gut microbiota on phylum level for recently fed fish, starved fish (48 h feed withdrawal) and environmental control samples (feed and marine water (MW)).

## Data Availability

The raw 16S rRNA gene sequence files and metadata are deposited at the NCBI SRA database under the BioProject PRJNA866155. The datasets generated during and/or analysed during the current study are available from the corresponding author on reasonable request.

## Abbreviations

AGD: Amoebic gill disease
AS: Aerobic scope
F: Farmed
FEAST: Fast expectation–maximization for microbial source tracking
HFF: Hybrid farmed female
HPRA: Health Products Regulatory Authority
HWF: Hybrid wild female
MG: Midgut
MMR: Maximum metabolic rate
OTU: Operational taxonomic unit
PC: Pyloric caeca
PCoA: Principal Coordinate Analysis
PCR: Polymerase Chain Reaction
PERMANOVA: Permutational Analysis of Variance
PIT tags: Passive Integrated Transponder
W: Wild
