# Supplementary material for: Links between host genetics, metabolism, gut microbiome and amoebic gill disease (AGD) in Atlantic salmon
Source: Anim Microbiome. 2022 Sep 15;4:53. doi: 10.1186/s42523-022-00203-x (PMC9479442; doi:10.1186/s42523-022-00203-x)
Supplement: Supplementary file 1 — Additional file 1: Table S1. Overview of the metabolic rate experiment. Sample_Id refers to individual fish. Sampling day refers to the day when fish were caught. Processing day refers to the day fish were dissected. Experiment day equals processing days, only in another format. Temperature refers to the water temperature at the sea pens on each sampling day. Table S2. First round PCR primers used for NGS library preparation. Table S3. Second round PCR primers used for NGS library preparation. Table S4. Mean length, weight and Fulton’s condition factor per genetic origin for 3 points in time (pre sea pen hatchery, date of sea pen transfer and at the termination point of the experiment). Table S5. ANCOVA results of the effect of AGD severity Score on weight adjusted SMR (left), MMR (middle) and AS (right) measures. Significance codes: *** < 0.001; ** < 0.01; * < 0.05. Table S6. Multiple comparisons of means of metabolic rate measures between different AGD severity scores (Tukey post-hoc for weight-adjusted model). Significance codes: ** < 0.01; * < 0.05. Table S7. Permanova results showing differences between gut microbial community compositions of starved and fed fish for two different timepoints T0 and T1 in PC samples. Significance codes: ** < 0.01; * < 0.05. Table S8. Permanova results showing differences between gut microbial community compositions of starved and fed fish for two different timepoints T0 and T1 in MG samples. Significance codes: ** < 0.01; * < 0.05. Table S9. Linear model for Chao1 richness estimates in MG samples calculated by the interaction term of AGD severity score and genetic origin. AGD severity of 0 and farmed origin served as baseline for comparisons. Significance codes: *** < 0.001; ** < 0.01; * < 0.05. Table S10. Permutational analysis of variance (PERMANOVA) results testing the effect of AGD severity score, genetic origin and processing day (day of gut dissection) on pyloric caeca (PC) microbial community composition based on weighted [file 42523_2022_203_MOESM1_ESM.docx]

Additional file 1

**Supplementary Tables**

Additional file 1: Table S1. Overview of the metabolic rate experiment. Sample_Id refers to individual fish. Sampling day refers to the day when fish were caught. Processing day refers to the day fish were dissected. Experiment day equals processing days, only in another format. Temperature refers to the water temperature at the sea pens on each sampling day.

| Sample_ID | Sampling Day | Processing Day | Experiment Day | Temperature [°C] |
| --- | --- | --- | --- | --- |
| H18D_1000 | 11/06/2019 | 14/06/2019 | Day0 | 14.17 |
| H18D_1001 | 11/06/2019 | 14/06/2019 | Day0 | 14.17 |
| H18D_1002 | 11/06/2019 | 14/06/2019 | Day0 | 14.17 |
| H18D_1003 | 11/06/2019 | 14/06/2019 | Day0 | 14.17 |
| H18D_1004 | 11/06/2019 | 14/06/2019 | Day0 | 14.17 |
| H18D_1005 | 11/06/2019 | 14/06/2019 | Day0 | 14.17 |
| H18D_1006 | 11/06/2019 | 14/06/2019 | Day0 | 14.17 |
| H18D_1007 | 11/06/2019 | 14/06/2019 | Day0 | 14.17 |
| H18D_1008 | 14/06/2019 | 17/06/2019 | Day03 | 13.62 |
| H18D_1009 | 14/06/2019 | 17/06/2019 | Day03 | 13.62 |
| H18D_1010 | 14/06/2019 | 17/06/2019 | Day03 | 13.62 |
| H18D_1011 | 14/06/2019 | 17/06/2019 | Day03 | 13.62 |
| H18D_1012 | 14/06/2019 | 17/06/2019 | Day03 | 13.62 |
| H18D_1013 | 14/06/2019 | 17/06/2019 | Day03 | 13.62 |
| H18D_1014 | 14/06/2019 | 17/06/2019 | Day03 | 13.62 |
| H18D_1015 | 14/06/2019 | 17/06/2019 | Day03 | 13.62 |
| H18D_1016 | 18/06/2019 | 21/06/2019 | Day07 | 14.22 |
| H18D_1017 | 18/06/2019 | 21/06/2019 | Day07 | 14.22 |
| H18D_1018 | 18/06/2019 | 21/06/2019 | Day07 | 14.22 |
| H18D_1019 | 18/06/2019 | 21/06/2019 | Day07 | 14.22 |
| H18D_1020 | 18/06/2019 | 21/06/2019 | Day07 | 14.22 |
| H18D_1021 | 18/06/2019 | 21/06/2019 | Day07 | 14.22 |
| H18D_1022 | 18/06/2019 | 21/06/2019 | Day07 | 14.22 |
| H18D_1023 | 21/06/2019 | 24/06/2019 | Day10 | 14.83 |
| H18D_1024 | 21/06/2019 | 24/06/2019 | Day10 | 14.83 |
| H18D_1025 | 21/06/2019 | 24/06/2019 | Day10 | 14.83 |
| H18D_1026 | 21/06/2019 | 24/06/2019 | Day10 | 14.83 |
| H18D_1027 | 21/06/2019 | 24/06/2019 | Day10 | 14.83 |
| H18D_1028 | 21/06/2019 | 24/06/2019 | Day10 | 14.83 |
| H18D_1029 | 21/06/2019 | 24/06/2019 | Day10 | 14.83 |
| H18D_1030 | 21/06/2019 | 24/06/2019 | Day10 | 14.83 |
| H18D_1031 | 25/06/2019 | 28/06/2019 | Day14 | 14.95 |
| H18D_1032 | 25/06/2019 | 28/06/2019 | Day14 | 14.95 |
| H18D_1033 | 25/06/2019 | 28/06/2019 | Day14 | 14.95 |
| H18D_1034 | 25/06/2019 | 28/06/2019 | Day14 | 14.95 |
| H18D_1035 | 25/06/2019 | 28/06/2019 | Day14 | 14.95 |
| H18D_1036 | 25/06/2019 | 28/06/2019 | Day14 | 14.95 |
| H18D_1037 | 25/06/2019 | 28/06/2019 | Day14 | 14.95 |
| H18D_1038 | 25/06/2019 | 28/06/2019 | Day14 | 14.95 |
| H18D_1039 | 12/07/2019 | 14/07/2019 | Day31 | 16.53 |
| H18D_1040 | 12/07/2019 | 14/07/2019 | Day31 | 16.53 |
| H18D_1041 | 12/07/2019 | 14/07/2019 | Day31 | 16.53 |
| H18D_1042 | 12/07/2019 | 14/07/2019 | Day31 | 16.53 |
| H18D_1043 | 12/07/2019 | 14/07/2019 | Day31 | 16.53 |
| H18D_1044 | 12/07/2019 | 14/07/2019 | Day31 | 16.53 |
| H18D_1045 | 12/07/2019 | 14/07/2019 | Day31 | 16.53 |
| H18D_1046 | 12/07/2019 | 15/07/2019 | Day32 | 16.53 |
| H18D_1047 | 12/07/2019 | 15/07/2019 | Day32 | 16.53 |
| H18D_1048 | 12/07/2019 | 15/07/2019 | Day32 | 16.53 |
| H18D_1049 | 12/07/2019 | 15/07/2019 | Day32 | 16.53 |
| H18D_1050 | 12/07/2019 | 15/07/2019 | Day32 | 16.53 |
| H18D_1051 | 12/07/2019 | 15/07/2019 | Day32 | 16.53 |
| H18D_1052 | 16/07/2019 | 18/07/2019 | Day35 | 17.13 |
| H18D_1053 | 16/07/2019 | 18/07/2019 | Day35 | 17.13 |
| H18D_1054 | 16/07/2019 | 18/07/2019 | Day35 | 17.13 |
| H18D_1055 | 16/07/2019 | 18/07/2019 | Day35 | 17.13 |
| H18D_1056 | 16/07/2019 | 18/07/2019 | Day35 | 17.13 |
| H18D_1057 | 16/07/2019 | 18/07/2019 | Day35 | 17.13 |
| H18D_1058 | 16/07/2019 | 18/07/2019 | Day35 | 17.13 |
| H18D_1059 | 16/07/2019 | 18/07/2019 | Day35 | 17.13 |
| H18D_1060 | 16/07/2019 | 18/07/2019 | Day35 | 17.13 |
| H18D_1061 | 16/07/2019 | 19/07/2019 | Day36 | 17.13 |
| H18D_1062 | 16/07/2019 | 19/07/2019 | Day36 | 17.13 |
| H18D_1063 | 16/07/2019 | 19/07/2019 | Day36 | 17.13 |
| H18D_1064 | 16/07/2019 | 19/07/2019 | Day36 | 17.13 |
| H18D_1065 | 16/07/2019 | 19/07/2019 | Day36 | 17.13 |
| H18D_1066 | 16/07/2019 | 19/07/2019 | Day36 | 17.13 |
| H18D_1067 | 16/07/2019 | 19/07/2019 | Day36 | 17.13 |
| H18D_1068 | 16/07/2019 | 19/07/2019 | Day36 | 17.13 |
| H18D_1069 | 19/07/2019 | 21/07/2019 | Day38 | 17.31 |
| H18D_1070 | 19/07/2019 | 21/07/2019 | Day38 | 17.31 |
| H18D_1071 | 19/07/2019 | 21/07/2019 | Day38 | 17.31 |
| H18D_1072 | 19/07/2019 | 21/07/2019 | Day38 | 17.31 |
| H18D_1073 | 19/07/2019 | 21/07/2019 | Day38 | 17.31 |
| H18D_1074 | 19/07/2019 | 21/07/2019 | Day38 | 17.31 |
| H18D_1075 | 19/07/2019 | 21/07/2019 | Day38 | 17.31 |
| H18D_1076 | 19/07/2019 | 21/07/2019 | Day38 | 17.31 |
| H18D_1077 | 19/07/2019 | 22/07/2019 | Day39 | 17.31 |
| H18D_1078 | 19/07/2019 | 22/07/2019 | Day39 | 17.31 |
| H18D_1079 | 19/07/2019 | 22/07/2019 | Day39 | 17.31 |
| H18D_1080 | 19/07/2019 | 22/07/2019 | Day39 | 17.31 |
| H18D_1081 | 19/07/2019 | 22/07/2019 | Day39 | 17.31 |
| H18D_1082 | 19/07/2019 | 22/07/2019 | Day39 | 17.31 |
| H18D_1083 | 19/07/2019 | 22/07/2019 | Day39 | 17.31 |
| H18D_1084 | 19/07/2019 | 22/07/2019 | Day39 | 17.31 |
| H18D_1085 | 23/07/2019 | 25/07/2019 | Day42 | 17.73 |
| H18D_1086 | 23/07/2019 | 25/07/2019 | Day42 | 17.73 |
| H18D_1087 | 23/07/2019 | 25/07/2019 | Day42 | 17.73 |
| H18D_1088 | 23/07/2019 | 25/07/2019 | Day42 | 17.73 |
| H18D_1089 | 23/07/2019 | 25/07/2019 | Day42 | 17.73 |
| H18D_1090 | 23/07/2019 | 25/07/2019 | Day42 | 17.73 |
| H18D_1091 | 23/07/2019 | 25/07/2019 | Day42 | 17.73 |
| H18D_1092 | 23/07/2019 | 25/07/2019 | Day42 | 17.73 |
| H18D_1093 | 23/07/2019 | 26/07/2019 | Day43 | 17.73 |
| H18D_1094 | 23/07/2019 | 26/07/2019 | Day43 | 17.73 |
| H18D_1095 | 23/07/2019 | 26/07/2019 | Day43 | 17.73 |
| H18D_1096 | 23/07/2019 | 26/07/2019 | Day43 | 17.73 |
| H18D_1097 | 23/07/2019 | 26/07/2019 | Day43 | 17.73 |
| H18D_1098 | 23/07/2019 | 26/07/2019 | Day43 | 17.73 |
| H18D_1099 | 23/07/2019 | 26/07/2019 | Day43 | 17.73 |
| H18D_1100 | 23/07/2019 | 26/07/2019 | Day43 | 17.73 |
| H18D_1101 | 26/07/2019 | 28/07/2019 | Day45 | 18.25 |
| H18D_1102 | 26/07/2019 | 28/07/2019 | Day45 | 18.25 |
| H18D_1103 | 26/07/2019 | 28/07/2019 | Day45 | 18.25 |
| H18D_1104 | 26/07/2019 | 28/07/2019 | Day45 | 18.25 |
| H18D_1105 | 26/07/2019 | 28/07/2019 | Day45 | 18.25 |
| H18D_1106 | 26/07/2019 | 28/07/2019 | Day45 | 18.25 |
| H18D_1107 | 26/07/2019 | 28/07/2019 | Day45 | 18.25 |
| H18D_1108 | 26/07/2019 | 28/07/2019 | Day45 | 18.25 |
| H18D_1109 | 26/07/2019 | 29/07/2019 | Day46 | 18.25 |
| H18D_1110 | 26/07/2019 | 29/07/2019 | Day46 | 18.25 |
| H18D_1111 | 26/07/2019 | 29/07/2019 | Day46 | 18.25 |
| H18D_1112 | 26/07/2019 | 29/07/2019 | Day46 | 18.25 |
| H18D_1113 | 26/07/2019 | 29/07/2019 | Day46 | 18.25 |
| H18D_1114 | 26/07/2019 | 29/07/2019 | Day46 | 18.25 |
| H18D_1115 | 26/07/2019 | 29/07/2019 | Day46 | 18.25 |
| H18D_1116 | 26/07/2019 | 29/07/2019 | Day46 | 18.25 |
| H18D_1117 | 26/07/2019 | 30/07/2019 | Day47 | 18.25 |
| H18D_1118 | 26/07/2019 | 30/07/2019 | Day47 | 18.25 |
| H18D_1119 | 26/07/2019 | 30/07/2019 | Day47 | 18.25 |
| H18D_1120 | 26/07/2019 | 30/07/2019 | Day47 | 18.25 |
| H18D_1121 | 26/07/2019 | 30/07/2019 | Day47 | 18.25 |
| H18D_1122 | 30/07/2019 | 01/08/2019 | Day49 | 18.26 |
| H18D_1123 | 30/07/2019 | 01/08/2019 | Day49 | 18.26 |
| H18D_1124 | 30/07/2019 | 01/08/2019 | Day49 | 18.26 |
| H18D_1125 | 30/07/2019 | 01/08/2019 | Day49 | 18.26 |
| H18D_1126 | 30/07/2019 | 01/08/2019 | Day49 | 18.26 |
| H18D_1127 | 30/07/2019 | 01/08/2019 | Day49 | 18.26 |
| H18D_1128 | 30/07/2019 | 01/08/2019 | Day49 | 18.26 |
| H18D_1129 | 30/07/2019 | 01/08/2019 | Day49 | 18.26 |
| H18D_1130 | 30/07/2019 | 02/08/2019 | Day50 | 18.26 |
| H18D_1131 | 30/07/2019 | 02/08/2019 | Day50 | 18.26 |
| H18D_1132 | 30/07/2019 | 02/08/2019 | Day50 | 18.26 |
| H18D_1133 | 30/07/2019 | 02/08/2019 | Day50 | 18.26 |
| H18D_1134 | 30/07/2019 | 02/08/2019 | Day50 | 18.26 |
| H18D_1135 | 30/07/2019 | 02/08/2019 | Day50 | 18.26 |
| H18D_1136 | 30/07/2019 | 02/08/2019 | Day50 | 18.26 |
| H18D_1137 | 30/07/2019 | 02/08/2019 | Day50 | 18.26 |
| H18D_1138 | 30/07/2019 | 03/08/2019 | Day50 | 18.26 |
| H18D_1139 | 30/07/2019 | 03/08/2019 | Day50 | 18.26 |
| H18D_1140 | 30/07/2019 | 03/08/2019 | Day50 | 18.26 |
| H18D_1141 | 30/07/2019 | 03/08/2019 | Day50 | 18.26 |
| H18D_1142 | 30/07/2019 | 03/08/2019 | Day50 | 18.26 |
| H18D_1143 | 30/07/2019 | 03/08/2019 | Day50 | 18.26 |
| H18D_1144 | 30/07/2019 | 03/08/2019 | Day50 | 18.26 |
| H18D_1145 | 30/07/2019 | 03/08/2019 | Day50 | 18.26 |

Additional file 1: Table S2. First round PCR primers used for NGS library preparation

| Primer | Label | Illumina 5′ sequencing primer (CS1/CS2) | Internal index length | Heterogeneity spacer | spacer 16S rRNA gene v1 primer |
| --- | --- | --- | --- | --- | --- |
| Forward | 27F | ACACTCTTTCCCTACACGACGCTCTTCCGATCT | 8bp | NNNN or NNN (5/3bp) | AGAGTTTGATCMTGGCTCAG |
| Reverse | 338R | GTGACTGGAGTTCAGACGTGTGCTCTTCCGATCT | 8bp | none | GCTGCCTCCCGTAGGAGT |

Additional file 1: Table S3. Second round PCR primers used for NGS library preparation

| Primer | Illumina 3′ flow cell linker (i5/i7) | External index length | Illumina 5′ sequencing primer (CS1/CS2) |
| --- | --- | --- | --- |
| Forward | AATGATACGGCG ACCACCGAGATC TACAC | 8bp | CACTCTTTCCCTACACGAC GCT |
| Reverse | CAAGCAGAAGAC GGCATACGAGAT | 8bp | GTGACTGGAGTTCAGACG TGTGCTC |

Additional file 1: Table S4. Mean length, weight and Fulton’s condition factor per genetic origin for 3 points in time (pre sea pen hatchery, date of sea pen transfer and at the termination point of the experiment)

| Points in time | Origin | Length (mm) +/- SD | Weight (g) +/-SD | Fulton’s K |
| --- | --- | --- | --- | --- |
| Hatchery  (27.03.2019) | Farmed  Hybrid  Wild | 200.10 +/- 19.07  175.64 +/- 13.12  160.90 +/- 11.50 | 94.45 +/- 25.12  61.84 +/- 14.37  46.18 +/- 10.23 | 1.17  1.14  1.10 |
| Sea Pen Transfer (09.05.2019) | Farmed  Hybrid  Wild | 235.70 +/- 18.07  191.85 +/- 23.78  173.00 +/- 25.51 | 137.60 +/- 28.67  78.04 +/- 21.08  56.28 +/- 21.38 | 1.05  1.10  1.08 |
| End of experiment  (30.07.2019) | Farmed  Hybrid  Wild | 246.34 +/- 18.75  214.46 +/- 16.13  204.55 +/- 12.81 | 135.44 +/- 37.50  84.76 +/- 21.69  70.35 +/- 14.36 | 0.90  0.85  0.82 |

Additional file 1: Table S5. ANCOVA results of the effect of AGD severity Score on weight adjusted SMR (left), MMR (middle) and AS (right) measures. Significance codes: ***< 0.001; **< 0.01; *< 0.05.

|  |  | SMR |  |  | MMR |  |  | AS |  |
| --- | --- | --- | --- | --- | --- | --- | --- | --- | --- |
|  | Sum Sq | F value | Sign. | Sum Sq | F value | Sign. | Sum Sq | F value | Sign. |
| (Intercept) | 7.4503 | 1060.758 | *** | 14.344 | 1125.618 | *** | 12.4814 | 602.15 | *** |
| log.weight | 1.9933 | 283.796 | *** | 1.8778 | 147.355 | *** | 1.7176 | 82.865 | *** |
| AGDScore | 0.1557 | 7.388 | *** | 0.1476 | 3.8611 | * | 0.1409 | 2.266 |  |
| Residuals | 0.8288 |  |  | 1.5547 |  |  | 2.5081 |  |  |

Additional file 1: Table S6. Multiple comparisons of means of metabolic rate measures between different AGD severity scores (Tukey post-hoc for weight-adjusted model). Significance codes: **< 0.01; *< 0.05.

|  |  | SMR |  |  | MMR |  |  | AS |  |
| --- | --- | --- | --- | --- | --- | --- | --- | --- | --- |
| Coefficients | Estimate | Pr(>\|t\|) | Sign. | Estimate | Pr(>\|t\|) | Sign. | Estimate | Pr(>\|t\|) | Sign. |
| AGD 0: AGD 1 | -0.0252 | 0.688 |  | 0.0087 | 0.991 |  | 0.0240 | 0.924 |  |
| AGD 0: AGD 2 | -0.0872 | 0.002 | ** | -0.0695 | 0.132 |  | -0.0553 | 0.523 |  |
| AGD 0: AGD 3 | -0.0765 | 0.002 | ** | -0.0745 | 0.033 | * | -0.0722 | 0.159 |  |
| AGD 1: AGD 2 | -0.0622 | 0.170 |  | -0.0783 | 0.203 |  | -0.0794 | 0.399 |  |
| AGD 1: AGD 3 | -0.0322 | 0.636 |  | -0.0832 | 0.099 |  | -0.0963 | 0.158 |  |
| AGD 2: AGD 3 | 0.0299 | 0.713 |  | -0.0049 | 0.999 |  | -0.0169 | 0.984 |  |

Additional file 1: Table S7. Permanova results showing differences between gut microbial community compositions of starved and fed fish for two different timepoints T0 and T1 in PC samples. Significance codes: **< 0.01; *< 0.05.

| Groups | F | R2 | p.value | p.adjusted | Significance |
| --- | --- | --- | --- | --- | --- |
| T0Fed vs T1Fed | 1.817 | 0.132 | 0.078 | 0.094 |  |
| T0Fed vs T0Starved | 12.588 | 0.473 | 0.001 | 0.003 | ** |
| T0Fed vs T1Starved | 7.932 | 0.398 | 0.001 | 0.003 | ** |
| T1Fed vs T0Starved | 10.960 | 0.477 | 0.002 | 0.004 | ** |
| T1Fed vs T1Starved | 6.944 | 0.410 | 0.003 | 0.005 | ** |
| T0Starved vs T1Starved | 0.973 | 0.075 | 0.489 | 0.489 |  |

Additional file 1: Table S8. Permanova results showing differences between gut microbial community compositions of starved and fed fish for two different timepoints T0 and T1 in MG samples. Significance codes: **< 0.01; *< 0.05.

| Groups | F | R2 | p.value | p.adjusted | Significance |
| --- | --- | --- | --- | --- | --- |
| T0Fed vs T1Fed | 2.208 | 0.145 | 0.024 | 0.029 | * |
| T0Fed vs T0Starved | 11.593 | 0.436 | 0.001 | 0.002 | ** |
| T0Fed vs T1Starved | 7.975 | 0.380 | 0.001 | 0.002 | ** |
| T1Fed vs T0Starved | 13.785 | 0.535 | 0.001 | 0.002 | ** |
| T1Fed vs T1Starved | 10.265 | 0.507 | 0.006 | 0.009 | ** |
| T0Starved vs T1Starved | 1.303 | 0.098 | 0.276 | 0.276 |  |

Additional file 1: Table S9. Linear model for Chao1 richness estimates in MG samples calculated by the interaction term of AGD severity score and genetic origin. AGD severity of 0 and farmed origin served as baseline for comparisons. Significance codes: ***< 0.001; **< 0.01; *< 0.05.

| Coefficients | Estimate | Std. Error | t value | Pr(>\|t\|) |  |
| --- | --- | --- | --- | --- | --- |
| (Intercept) | 324.71 | 16.396 | 19.804 | < 2e-16 | *** |
| AGDScore 1 | 3.968 | 44.903 | 0.088 | 0.92974 |  |
| AGDScore 2 | -75.249 | 50.978 | -1.476 | 0.14253 |  |
| AGDScore 3 | -158.505 | 50.978 | -3.109 | 0.00234 | ** |
| Origin HFF | -57.854 | 24.219 | -2.389 | 0.01846 | * |
| Origin HWF | -37.119 | 26.565 | -1.397 | 0.1649 |  |
| Origin W | -54.733 | 23.932 | -2.287 | 0.02395 | * |
| AGDScore 1: Origin HFF | -18.122 | 61.089 | -0.297 | 0.76725 |  |
| AGDScore 2: Origin HFF | 88.655 | 80.071 | 1.107 | 0.27042 |  |
| AGDScore 3: Origin HFF | 83.501 | 62.57 | 1.335 | 0.18456 |  |
| AGDScore 1: Origin HWF | -27.902 | 77.123 | -0.362 | 0.71815 |  |
| AGDScore 2: Origin HWF | 11.971 | 64.812 | 0.185 | 0.85377 |  |
| AGDScore 3: Origin HWF | 187.101 | 69.159 | 2.705 | 0.00782 | ** |
| AGDScore 1: Origin W | -75.08 | 68.191 | -1.101 | 0.27309 |  |
| AGDScore 2: Origin W | -13.624 | 65.579 | -0.208 | 0.83577 |  |
| AGDScore 3: Origin W | 136.461 | 65.579 | 2.081 | 0.03957 | * |

Additional file 1: Table S10. Permutational analysis of variance (PERMANOVA) results testing the effect of AGD severity score, genetic origin and processing day (day of gut dissection) on pyloric caeca (PC) microbial community composition based on weighted unifrac distance matrices.

|  | Df | SumsOfSqs | MeanSqs | F.Model | R^2^ | Pr(>F) | Significance |
| --- | --- | --- | --- | --- | --- | --- | --- |
| Origin | 3 | 0.3681 | 0.12268 | 1.3178 | 0.02693 | 0.1597 |  |
| AGDScore | 3 | 1.2882 | 0.42941 | 4.6123 | 0.09426 | 0.0001 | *** |
| ProcessingDay | 17 | 3.4451 | 0.20265 | 2.1767 | 0.25208 | 0.0001 | *** |
| Residuals | 92 | 8.5652 | 0.0931 |  | 0.62672 |  |  |
| Total | 115 | 13.6666 |  |  | 1 |  |  |

Additional file 1: Table S11. Permanova of pairwise comparisons of PC samples grouped by day of their processing. Distance matrix calculated by weighted UniFrac measure. Permutations used: 9999. Significance codes: ***< 0.001; **< 0.01; *< 0.05.

| Groups | F | R2 | p.value | p.adjusted | Significance |
| --- | --- | --- | --- | --- | --- |
|  |  |  |  |  |  |
| Day0 vs Day03 | 1.5352 | 0.1225 | 0.143 | 0.179 |  |
| Day0 vs Day07 | 0.9736 | 0.0750 | 0.465 | 0.474 |  |
| Day0 vs Day10 | 2.4742 | 0.1502 | 0.049 | 0.084 |  |
| Day0 vs Day14 | 1.1710 | 0.0772 | 0.324 | 0.344 |  |
| Day0 vs Day31 | 2.3879 | 0.1552 | 0.033 | 0.070 |  |
| Day0 vs Day32 | 2.7732 | 0.2013 | 0.047 | 0.082 |  |
| Day0 vs Day35 | 3.0856 | 0.1918 | 0.012 | 0.047 | * |
| Day0 vs Day36 | 1.0803 | 0.0767 | 0.336 | 0.352 |  |
| Day0 vs Day38 | 3.9314 | 0.2192 | 0.002 | 0.045 | * |
| Day0 vs Day39 | 1.9212 | 0.1207 | 0.112 | 0.149 |  |
| Day0 vs Day42 | 3.0127 | 0.1771 | 0.015 | 0.048 | * |
| Day0 vs Day43 | 4.9092 | 0.2903 | 0.008 | 0.045 | * |
| Day0 vs Day45 | 4.3044 | 0.3235 | 0.016 | 0.050 | * |
| Day0 vs Day46 | 5.7595 | 0.3243 | 0.001 | 0.045 | * |
| Day0 vs Day49 | 4.5736 | 0.2760 | 0.003 | 0.045 | * |
| Day0 vs Day50 | 2.9574 | 0.2119 | 0.013 | 0.047 | * |
| Day0 vs Day51 | 4.1226 | 0.2726 | 0.011 | 0.045 | * |
| Day03 vs Day07 | 1.5755 | 0.1490 | 0.073 | 0.115 |  |
| Day03 vs Day10 | 2.5267 | 0.1868 | 0.041 | 0.077 |  |
| Day03 vs Day14 | 2.2803 | 0.1717 | 0.051 | 0.087 |  |
| Day03 vs Day31 | 2.5915 | 0.2058 | 0.037 | 0.074 |  |
| Day03 vs Day32 | 4.9486 | 0.3822 | 0.014 | 0.048 | * |
| Day03 vs Day35 | 4.5657 | 0.3135 | 0.005 | 0.045 | * |
| Day03 vs Day36 | 1.4466 | 0.1264 | 0.220 | 0.250 |  |
| Day03 vs Day38 | 4.3128 | 0.2816 | 0.015 | 0.048 | * |
| Day03 vs Day39 | 2.0583 | 0.1576 | 0.097 | 0.135 |  |
| Day03 vs Day42 | 3.8761 | 0.2606 | 0.010 | 0.045 | * |
| Day03 vs Day43 | 4.0509 | 0.3104 | 0.037 | 0.074 |  |
| Day03 vs Day45 | 5.1336 | 0.4611 | 0.008 | 0.045 | * |
| Day03 vs Day46 | 5.2137 | 0.3668 | 0.002 | 0.045 | * |
| Day03 vs Day49 | 5.7744 | 0.3908 | 0.013 | 0.047 | * |
| Day03 vs Day50 | 3.8175 | 0.3230 | 0.012 | 0.047 | * |
| Day03 vs Day51 | 5.8675 | 0.4231 | 0.009 | 0.045 | * |
| Day07 vs Day10 | 1.6207 | 0.1190 | 0.149 | 0.181 |  |
| Day07 vs Day14 | 1.5978 | 0.1175 | 0.131 | 0.168 |  |
| Day07 vs Day31 | 2.8324 | 0.2048 | 0.031 | 0.070 |  |
| Day07 vs Day32 | 3.7058 | 0.2917 | 0.028 | 0.070 |  |
| Day07 vs Day35 | 2.2914 | 0.1724 | 0.033 | 0.070 |  |
| Day07 vs Day36 | 1.0918 | 0.0903 | 0.348 | 0.362 |  |
| Day07 vs Day38 | 2.5783 | 0.1769 | 0.039 | 0.076 |  |
| Day07 vs Day39 | 1.7671 | 0.1284 | 0.118 | 0.154 |  |
| Day07 vs Day42 | 2.2628 | 0.1587 | 0.033 | 0.070 |  |
| Day07 vs Day43 | 2.9900 | 0.2302 | 0.030 | 0.070 |  |
| Day07 vs Day45 | 2.8454 | 0.2890 | 0.018 | 0.053 |  |
| Day07 vs Day46 | 3.7716 | 0.2739 | 0.003 | 0.045 | * |
| Day07 vs Day49 | 3.8465 | 0.2778 | 0.009 | 0.045 | * |
| Day07 vs Day50 | 3.1367 | 0.2584 | 0.008 | 0.045 | * |
| Day07 vs Day51 | 4.3731 | 0.3270 | 0.008 | 0.045 | * |
| Day10 vs Day14 | 1.4252 | 0.0924 | 0.200 | 0.230 |  |
| Day10 vs Day31 | 1.9501 | 0.1304 | 0.144 | 0.179 |  |
| Day10 vs Day32 | 3.0422 | 0.2166 | 0.046 | 0.081 |  |
| Day10 vs Day35 | 2.9741 | 0.1862 | 0.033 | 0.070 |  |
| Day10 vs Day36 | 1.6349 | 0.1117 | 0.167 | 0.197 |  |
| Day10 vs Day38 | 3.0795 | 0.1803 | 0.025 | 0.068 |  |
| Day10 vs Day39 | 1.7370 | 0.1104 | 0.149 | 0.181 |  |
| Day10 vs Day42 | 2.5770 | 0.1555 | 0.040 | 0.077 |  |
| Day10 vs Day43 | 2.1912 | 0.1544 | 0.092 | 0.129 |  |
| Day10 vs Day45 | 2.4604 | 0.2147 | 0.077 | 0.118 |  |
| Day10 vs Day46 | 3.3144 | 0.2164 | 0.011 | 0.045 | * |
| Day10 vs Day49 | 4.0095 | 0.2504 | 0.007 | 0.045 | * |
| Day10 vs Day50 | 2.8555 | 0.2061 | 0.031 | 0.070 |  |
| Day10 vs Day51 | 3.5398 | 0.2435 | 0.027 | 0.069 |  |
| Day14 vs Day31 | 1.0922 | 0.0775 | 0.298 | 0.326 |  |
| Day14 vs Day32 | 1.2409 | 0.1014 | 0.221 | 0.250 |  |
| Day14 vs Day35 | 2.6096 | 0.1672 | 0.045 | 0.080 |  |
| Day14 vs Day36 | 1.1270 | 0.0798 | 0.268 | 0.297 |  |
| Day14 vs Day38 | 3.9922 | 0.2219 | 0.013 | 0.047 | * |
| Day14 vs Day39 | 0.8313 | 0.0561 | 0.416 | 0.427 |  |
| Day14 vs Day42 | 2.2595 | 0.1390 | 0.078 | 0.118 |  |
| Day14 vs Day43 | 3.6594 | 0.2337 | 0.027 | 0.069 |  |
| Day14 vs Day45 | 3.3503 | 0.2713 | 0.019 | 0.054 |  |
| Day14 vs Day46 | 3.9976 | 0.2499 | 0.006 | 0.045 | * |
| Day14 vs Day49 | 2.6480 | 0.1808 | 0.053 | 0.088 |  |
| Day14 vs Day50 | 1.5705 | 0.1249 | 0.146 | 0.180 |  |
| Day14 vs Day51 | 1.7792 | 0.1392 | 0.121 | 0.157 |  |
| Day31 vs Day32 | 0.7709 | 0.0716 | 0.613 | 0.617 |  |
| Day31 vs Day35 | 3.8317 | 0.2420 | 0.032 | 0.070 |  |
| Day31 vs Day36 | 1.2211 | 0.0924 | 0.280 | 0.308 |  |
| Day31 vs Day38 | 5.3988 | 0.2934 | 0.010 | 0.045 | * |
| Day31 vs Day39 | 1.1236 | 0.0796 | 0.313 | 0.337 |  |
| Day31 vs Day42 | 3.5564 | 0.2148 | 0.032 | 0.070 |  |
| Day31 vs Day43 | 3.7913 | 0.2563 | 0.039 | 0.076 |  |
| Day31 vs Day45 | 4.1483 | 0.3415 | 0.009 | 0.045 | * |
| Day31 vs Day46 | 5.0366 | 0.3141 | 0.006 | 0.045 | * |
| Day31 vs Day49 | 3.9833 | 0.2658 | 0.024 | 0.067 |  |
| Day31 vs Day50 | 1.5790 | 0.1364 | 0.116 | 0.153 |  |
| Day31 vs Day51 | 1.8778 | 0.1581 | 0.080 | 0.119 |  |
| Day32 vs Day35 | 5.4287 | 0.3519 | 0.017 | 0.052 |  |
| Day32 vs Day36 | 2.0729 | 0.1717 | 0.101 | 0.139 |  |
| Day32 vs Day38 | 7.0285 | 0.3899 | 0.007 | 0.045 | * |
| Day32 vs Day39 | 1.6173 | 0.1282 | 0.183 | 0.214 |  |
| Day32 vs Day42 | 4.7344 | 0.3009 | 0.007 | 0.045 | * |
| Day32 vs Day43 | 5.8827 | 0.3953 | 0.026 | 0.069 |  |
| Day32 vs Day45 | 6.6813 | 0.5269 | 0.015 | 0.048 | * |
| Day32 vs Day46 | 6.5139 | 0.4199 | 0.004 | 0.045 | * |
| Day32 vs Day49 | 4.1401 | 0.3151 | 0.041 | 0.077 |  |
| Day32 vs Day50 | 1.0875 | 0.1197 | 0.265 | 0.296 |  |
| Day32 vs Day51 | 1.5661 | 0.1637 | 0.265 | 0.296 |  |
| Day35 vs Day36 | 2.3011 | 0.1609 | 0.080 | 0.119 |  |
| Day35 vs Day38 | 2.2100 | 0.1453 | 0.087 | 0.123 |  |
| Day35 vs Day39 | 2.5061 | 0.1616 | 0.075 | 0.116 |  |
| Day35 vs Day42 | 1.0879 | 0.0772 | 0.324 | 0.344 |  |
| Day35 vs Day43 | 2.8545 | 0.2060 | 0.035 | 0.073 |  |
| Day35 vs Day45 | 1.9966 | 0.1997 | 0.112 | 0.149 |  |
| Day35 vs Day46 | 3.4179 | 0.2371 | 0.006 | 0.045 | * |
| Day35 vs Day49 | 3.9837 | 0.2659 | 0.010 | 0.045 | * |
| Day35 vs Day50 | 4.1991 | 0.2957 | 0.011 | 0.045 | * |
| Day35 vs Day51 | 5.8217 | 0.3680 | 0.008 | 0.045 | * |
| Day36 vs Day38 | 3.3525 | 0.2050 | 0.033 | 0.070 |  |
| Day36 vs Day39 | 0.6851 | 0.0501 | 0.553 | 0.560 |  |
| Day36 vs Day42 | 2.2257 | 0.1462 | 0.084 | 0.122 |  |
| Day36 vs Day43 | 2.6332 | 0.1931 | 0.081 | 0.119 |  |
| Day36 vs Day45 | 3.1445 | 0.2822 | 0.044 | 0.080 |  |
| Day36 vs Day46 | 3.8792 | 0.2607 | 0.005 | 0.045 | * |
| Day36 vs Day49 | 3.5907 | 0.2461 | 0.027 | 0.069 |  |
| Day36 vs Day50 | 2.2658 | 0.1847 | 0.036 | 0.074 |  |
| Day36 vs Day51 | 2.9654 | 0.2287 | 0.059 | 0.096 |  |
| Day38 vs Day39 | 3.9359 | 0.2194 | 0.019 | 0.054 |  |
| Day38 vs Day42 | 1.0716 | 0.0711 | 0.313 | 0.337 |  |
| Day38 vs Day43 | 2.2068 | 0.1553 | 0.085 | 0.123 |  |
| Day38 vs Day45 | 1.3900 | 0.1338 | 0.186 | 0.216 |  |
| Day38 vs Day46 | 3.7774 | 0.2394 | 0.004 | 0.045 | * |
| Day38 vs Day49 | 6.0716 | 0.3360 | 0.002 | 0.045 | * |
| Day38 vs Day50 | 5.5075 | 0.3336 | 0.007 | 0.045 | * |
| Day38 vs Day51 | 7.4306 | 0.4032 | 0.005 | 0.045 | * |
| Day39 vs Day42 | 2.2601 | 0.1390 | 0.062 | 0.099 |  |
| Day39 vs Day43 | 2.7851 | 0.1884 | 0.087 | 0.123 |  |
| Day39 vs Day45 | 2.9966 | 0.2498 | 0.054 | 0.089 |  |
| Day39 vs Day46 | 3.6074 | 0.2311 | 0.014 | 0.048 | * |
| Day39 vs Day49 | 2.9182 | 0.1956 | 0.061 | 0.098 |  |
| Day39 vs Day50 | 1.9330 | 0.1495 | 0.111 | 0.149 |  |
| Day39 vs Day51 | 2.3200 | 0.1742 | 0.111 | 0.149 |  |
| Day42 vs Day43 | 2.1847 | 0.1540 | 0.075 | 0.116 |  |
| Day42 vs Day45 | 1.6229 | 0.1528 | 0.152 | 0.182 |  |
| Day42 vs Day46 | 3.0323 | 0.2017 | 0.006 | 0.045 | * |
| Day42 vs Day49 | 3.8052 | 0.2408 | 0.011 | 0.045 | * |
| Day42 vs Day50 | 4.0408 | 0.2687 | 0.011 | 0.045 | * |
| Day42 vs Day51 | 5.1583 | 0.3192 | 0.005 | 0.045 | * |
| Day43 vs Day45 | 1.1901 | 0.1453 | 0.394 | 0.407 |  |
| Day43 vs Day46 | 1.5201 | 0.1320 | 0.165 | 0.196 |  |
| Day43 vs Day49 | 4.1499 | 0.2933 | 0.018 | 0.053 |  |
| Day43 vs Day50 | 3.7812 | 0.2958 | 0.032 | 0.070 |  |
| Day43 vs Day51 | 5.3108 | 0.3711 | 0.043 | 0.079 |  |
| Day45 vs Day46 | 1.1855 | 0.1448 | 0.328 | 0.346 |  |
| Day45 vs Day49 | 2.8385 | 0.2885 | 0.052 | 0.087 |  |
| Day45 vs Day50 | 3.0760 | 0.3389 | 0.045 | 0.080 |  |
| Day45 vs Day51 | 5.2185 | 0.4652 | 0.039 | 0.076 |  |
| Day46 vs Day49 | 1.7118 | 0.1462 | 0.142 | 0.179 |  |
| Day46 vs Day50 | 3.5315 | 0.2818 | 0.010 | 0.045 | * |
| Day46 vs Day51 | 4.6658 | 0.3414 | 0.014 | 0.048 | * |
| Day49 vs Day50 | 1.9563 | 0.1786 | 0.136 | 0.173 |  |
| Day49 vs Day51 | 2.2097 | 0.1971 | 0.150 | 0.181 |  |
| Day50 vs Day51 | 0.3347 | 0.0402 | 0.844 | 0.844 |  |

Additional file 1: Table S12. Permutational analysis of variance (PERMANOVA) results testing the effect of AGD severity score, genetic origin and processing day (day of gut dissection) on midgut (MG) microbial community composition based on weighted unifrac distance matrices.

| Groups | Df | SumOfSqs | R2 | F | Pr(>F) | Significance |
| --- | --- | --- | --- | --- | --- | --- |
| Origin | 3 | 0.4172 | 0.0279 | 1.5823 | 0.093 |  |
| AGDScore | 3 | 1.0738 | 0.0718 | 4.0724 | 0.001 | *** |
| ProcessingDay | 17 | 3.6207 | 0.2421 | 2.4233 | 0.001 | *** |
| Residual | 112 | 9.8436 | 0.6582 |  |  |  |
| Total | 135 | 14.9553 | 1 |  |  |  |

Additional file 1: Table S13. Permutational analysis of variance (PERMANOVA) testing pairwise comparisons for midgut (MG) samples from different AGD severity groups. Distance matrix calculated by weighted UniFrac measure. Permutations used: 9999. Significance codes: **p< 0.01; *p< 0.05

| Groups | F | R2 | p.value | p.adjusted | Significance |
| --- | --- | --- | --- | --- | --- |
| AGD0 vs AGD1 | 1.804173 | 0.0178978 | 0.1 | 0.12 |  |
| AGD0 vs AGD2 | 3.953787 | 0.0376717 | 0.008 | 0.024 | * |
| AGD0 vs AGD3 | 5.421323 | 0.0495454 | 0.001 | 0.006 | ** |
| AGD1 vs AGD2 | 1.060662 | 0.03649819 | 0.301 | 0.301 |  |
| AGD1 vs AGD3 | 3.204815 | 0.09369486 | 0.014 | 0.028 | * |
| AGD2 vs AGD3 | 2.742479 | 0.07672884 | 0.06 | 0.09 |  |
