# Supplementary material for: Links between host genetics, metabolism, gut microbiome and amoebic gill disease (AGD) in Atlantic salmon
Source: Anim Microbiome. 2022 Sep 15;4:53. doi: 10.1186/s42523-022-00203-x (PMC9479442; doi:10.1186/s42523-022-00203-x)
Supplement: Supplementary file 2 — Additional file 2: Fig. S1. Percent water content of fish from different origins during the sea pen experiments. Data derived from fish of mixed (4th) sentinel pen over the course of the experiment. Significance was determined by pairwise t-testing against fish from farmed origin. F = Farmed, HFF = Hybrid Farmed Female, HWF = Hybrid Wild Female, W = Wild, Significance codes: ***p < 0.001; **p < 0.01. Fig. S2. Stacked bar plot showing the mean relative abundance of gut microbiota on phylum level for recently fed fish, starved fish (48 h feed withdrawal) and environmental control samples (feed and marine water (MW)). [file 42523_2022_203_MOESM2_ESM.docx]

Additional file 2

**Supplementary Figures**

**
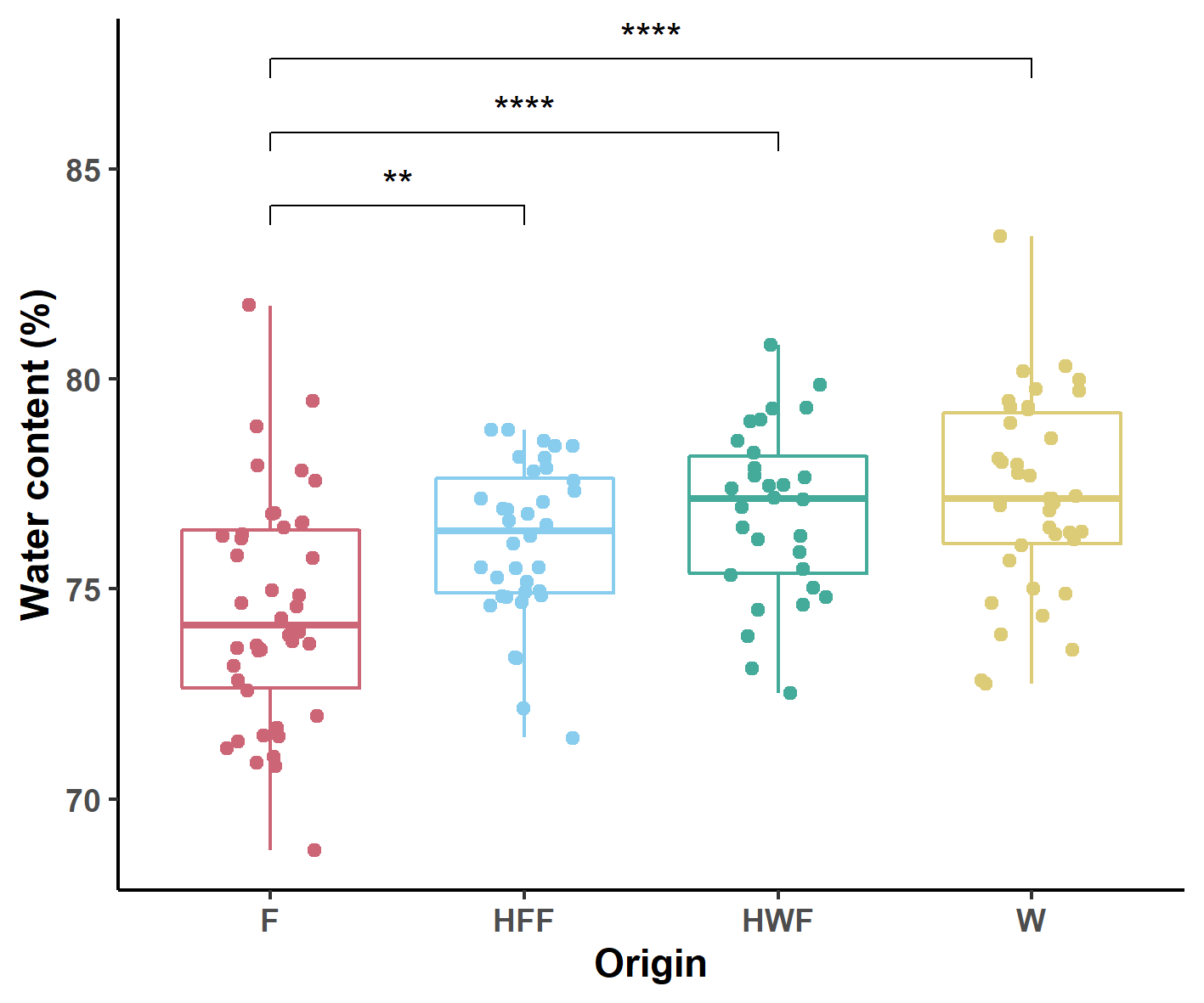
**

**Additional file 2: Fig. S1**. Percent water content of fish from different origins during the sea pen experiments. Data derived from fish of mixed (4^th^) sentinel pen over the course of the experiment. Significance was determined by pairwise t-testing against fish from farmed origin. F = Farmed, HFF = Hybrid Farmed Female, HWF = Hybrid Wild Female, W = Wild, Significance codes: ***p < 0.001; **p < 0.01.


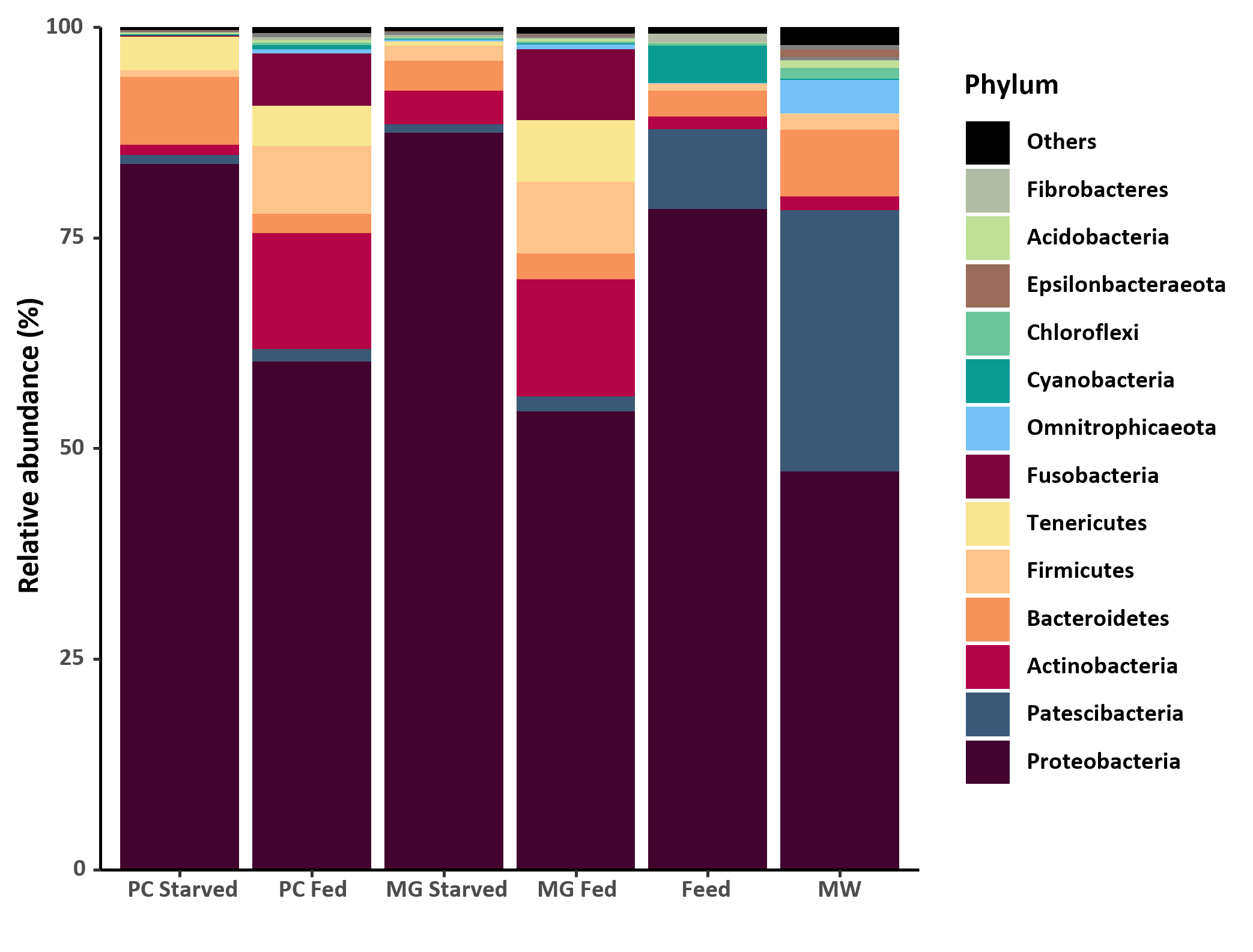
 **Additional file 2: Fig. S2**. Stacked bar plot showing the mean relative abundance of gut microbiota on phylum level for recently fed fish, starved fish (48 h feed withdrawal) and environmental control samples (feed and marine water (MW)).
